# Clathrin and AP2 Are Required for Phagocytic Receptor-Mediated Apoptotic Cell Clearance in *Caenorhabditis elegans*

**DOI:** 10.1371/journal.pgen.1003517

**Published:** 2013-05-16

**Authors:** Didi Chen, Youli Jian, Xuezhao Liu, Yuanya Zhang, Jingjing Liang, Xiaying Qi, Hongwei Du, Wei Zou, Lianwan Chen, Yongping Chai, Guangshuo Ou, Long Miao, Yingchun Wang, Chonglin Yang

**Affiliations:** 1 State Key Laboratory of Molecular Developmental Biology, Institute of Genetics and Developmental Biology, Chinese Academy of Sciences, Beijing, China; 2 Graduate School, Chinese Academy of Sciences, Beijing, China; 3 National Institute of Biological Sciences, Beijing, China; 4 Institute of Biophysics, Chinese Academy of Sciences, Beijing, China; The University of Dundee, United Kingdom

## Abstract

Clathrin and the multi-subunit adaptor protein complex AP2 are central players in clathrin-mediated endocytosis by which the cell selectively internalizes surface materials. Here, we report the essential role of clathrin and AP2 in phagocytosis of apoptotic cells. In *Caenorhabditis elegans*, depletion of the clathrin heavy chain CHC-1 and individual components of AP2 led to a significant accumulation of germ cell corpses, which resulted from defects in both cell corpse engulfment and phagosome maturation required for corpse removal. CHC-1 and AP2 components associate with phagosomes in an inter-dependent manner. Importantly, we found that the phagocytic receptor CED-1 interacts with the α subunit of AP2, while the CED-6/Gulp adaptor forms a complex with both CHC-1 and the AP2 complex, which likely mediates the rearrangement of the actin cytoskeleton required for cell corpse engulfment triggered by the CED-1 signaling pathway. In addition, CHC-1 and AP2 promote the phagosomal association of LST-4/Snx9/18/33 and DYN-1/dynamin by forming a complex with them, thereby facilitating the maturation of phagosomes necessary for corpse degradation. These findings reveal a non-classical role of clathrin and AP2 and establish them as indispensable regulators in phagocytic receptor-mediated apoptotic cell clearance.

## Author Summary

Phagocytosis of apoptotic cells is an indispensable part of the cell death program. During phagocytosis, the evolutionarily conserved CED-1 family phagocytic receptors recognize cell corpses and transduce engulfment signals to induce the formation and maturation of phagosomes. However, it remains largely unknown how the CED-1 signaling pathway induces the cytoskeletal reorganization required for corpse internalization and initiates phagosome maturation. Interestingly, cell corpse phagocytosis appears to resemble clathrin-mediated endocytosis (CME) of cell surface molecules in that both events cause receptor-dependent internalization of extracellular cargoes differing only in size. In CME, the recognition of plasma membrane receptors by adaptor proteins such as the AP2 complex triggers the formation of clathrin-coated vesicles (CCVs) with diameters ranging from 10–200 nm. Nevertheless, it is not known whether clathrin and AP2 also play a role in phagocytosis of apoptotic cells that are much larger than CCVs. Here we provide genetic, cell biological, and biochemical experimental findings to demonstrate that clathrin and AP2 act downstream of CED-1 to regulate the actin cytoskeleton rearrangement required for cell corpse internalization and cell corpse degradation. Clathrin and AP2 form two types of complex with factors required for engulfment and phagosome maturation. These findings establish clathrin and AP2 as essential players in phagocytic receptor-mediated apoptotic cell clearance.

## Introduction

Phagocytosis of apoptotic cells is critical to tissue remodeling, suppression of inflammation and control of immune responses [1], [2]. During phagocytosis, cell corpses are firstly engulfed and subsequently degraded by phagocytes, both phases being controlled by evolutionarily conserved regulators. In the lifetime of a *C. elegans* hermaphrodite, 131 somatic cells and about half the germ cells undergo apoptosis and the resulting cell corpses are quickly removed by neighboring cells in the soma or by sheath cells encasing the germ line. The engulfment of cell corpses is essentially controlled by two partially redundant signaling pathways that induce the cytoskeletal reorganization of engulfing cells [3]. In one pathway, the intracellular molecules CED-2/CrKII, CED-5/DOCK180, and CED-12/ELMO act through a protein interaction cascade to induce the activation of the small GTPase CED-10/Rac1, leading to the cytoskeleton reorganization necessary for engulfment [4]–[7]. In addition, the phosphatidylserine (Ptdser) receptor PSR-1 likely binds Ptdser, an “eat me” signal, and acts upstream of CED-2, -5, and -12 to regulate engulfment [4]. Two other signaling modules, INA-1/integrin-SRC-1/Src and UNC-73/TRIO-MIG-2/RhoG, were also found to function through the CED-5-CED-12 motility-promoting complex to facilitate CED-10 activation for corpse engulfment [8], [9]. In addition, a non-canonical Wnt pathway consisting of the MOM-5 receptor, GSK-3 kinase and APC/APR-1 may act through CED-2 to regulate CED-10 activity for cell corpse engulfment during early embryo development [10]. In the other pathway, the phagocytic receptor CED-1, which shares homology with the human scavenger receptor SREC, LRP/CD91 and MEGF10, and *Drosophila* Draper and Six-microns-under (SIMU) [11]–[15], recognizes apoptotic cells by interacting with TTR-52, a PtdSer-binding protein secreted from engulfing cells [16]. The adaptor protein CED-6/Gulp likely acts downstream of CED-1 to transduce engulfing signals to other effectors including the large GTPase DYN-1/dynamin, resulting in cell corpse engulfment and formation of phagosomes [14], [17], [18]. In addition, the ABC transporter CED-7 is also required for cell corpse recognition by CED-1 in embryos [11], [19]. Recent studies suggest that CED-7 acts with TTR-52 and NRF-5, another secreted PtdSer-binding protein, to mediate PtdSer transfer from cell corpses to phagocytes, thus promoting the recognition of cell corpses by CED-1 [20], [21]. Subsequent to corpse internalization, CED-1 is recycled from the phagosome back to the plasma membrane by the retromer complex [22]. Phagosomes enclosing cell corpses then undergo a maturation process by dynamically fusing with endocytic organelles including early and late endosomes as well as lysosomes, leading to formation of phagolysosomes in which cell corpses are ultimately digested. It has been found that several molecules required for endocytic trafficking, such as DYN-1/Dynamin, the phosphatidylinositol-3 kinase (PI3K) VPS-34, small GTPases and their regulators or effectors including RAB-2, RAB-5, TBC-2, RAB-7, RAB-14, and the HOPS complex, act in an ordered manner to regulate phagosome maturation [23]–[29]. As the phagolysosome forms, it is progressively acidified in order to activate lysosomal enzymes needed for cell corpse digestion [30].

The phagocytic receptor CED-1 plays a leading role in apoptotic cell clearance by recognizing cell corpses and transducing signals for engulfment and phagosome maturation. Nevertheless, it remains largely unknown how the CED-1-mediated signaling pathway triggers the cytoskeletal reorganization required for corpse internalization. In addition, the mechanisms governing the transition from corpse internalization to phagosome maturation are poorly understood. Interestingly, CED-1-mediated phagocytosis of cell corpses appears to resemble clathrin-mediated endocytosis (CME) of cell surface molecules in that both events cause receptor-dependent internalization of extracellular cargoes differing only in size [31]. In CME, recognition of the cytoplasmic domains of plasma membrane receptors by adaptor proteins triggers the formation of clathrin-coated vesicles (CCVs) with diameters ranging from 10–200 nm [32], [33]. The formation of cargo-containing CCVs requires several protein module-mediated events, including FCH domain-only (FCHO) complex-mediated initiation, adaptor protein 2 (AP2)-dependent cargo selection and coat building, dynamin-mediated scission, and auxilin- and heat shock cognate 70 (HSC70)-dependent uncoating [32]. Recent studies revealed that some of the molecules required for CME are involved in phagocytosis of pathogens or the maturation of phagosomes containing apoptotic cells. For example, clathrin and the adaptor protein Dab2 were found to be important for phagocytosis of pathogenic bacteria by mammalian cells [34]. In *C. elegans*, DYN-1/dynamin regulates the initiation of phagosome maturation [23]. However, whether other components of the CME pathway play a role in apoptotic cell clearance remains unknown.

In this study, we investigated the mechanisms underlying CED-1-mediated cytoskeleton reorganization for phagocytosis and uncovered the role of CME regulators in apoptotic cell clearance. Our findings revealed that clathrin and the AP2 complex, the central players in CME, are critical to apoptotic cell removal in *C. elegans*. We found that clathrin and AP2 act downstream of CED-1 and CED-6 by forming a complex with them, which mediates the rearrangement of the actin cytoskeleton required for cell corpse engulfment. In addition, we demonstrated that LST-4, the *C. elegans* homolog of Snx9/18/33, functions at an early step of phagosome maturation by promoting phagosomal association of DYN-1/dynamin. Furthermore, we demonstrated that clathrin and AP2 also interact with LST-4 and DYN-1 to regulate the initiation of phagosome maturation required for cell corpse degradation. These findings suggest that clathrin and AP2 play essential roles in the phagocytic receptor CED-1-mediated apoptotic cell clearance pathway by regulating cytoskeletal reorganization and facilitating phagosome maturation.

## Results

### Clathrin and the AP2 complex are important for engulfment of germ cell corpses in *C. elegans*

**Figure 1:**
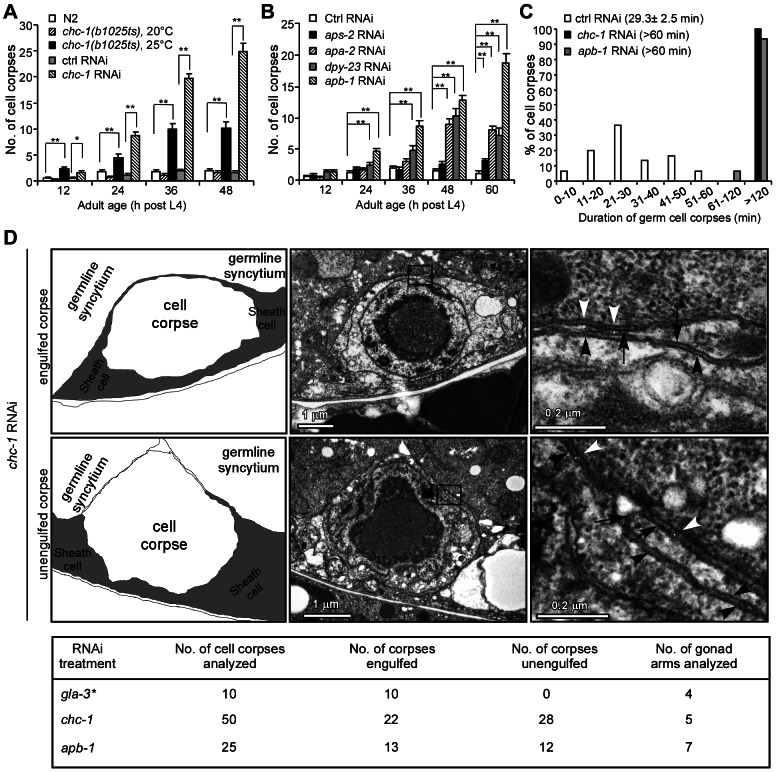
Clathrin and the AP2 complex are essential for apoptotic cell clearance. (A) Quantification of germ cell corpses in N2, *chc-1(b1025ts)*, and *chc-1(RNAi*) animals. Error bars represent SEM. * *p*<0.05, ** *p*<0.001. All other points had *p*>0.05. (B) Quantification of germ cell corpses in animals treated with RNAi of individual genes of the AP2 complex. Comparisons were performed between control (Ctrl) RNAi - and other RNAi treatments using unpaired *t*-tests. ** *p*<0.001. All other points had *p*>0.05. (C) Four-dimensional microscopy analysis of germ cell corpse duration in animals treated with Ctrl RNAi, *chc-1* RNAi and *apb-1* RNAi. 30 germ cell corpses were recorded for each RNAi treatment. Numbers in parenthesis indicate the average time of corpse duration (mean±SEM). (D) Representative transmission electron micrographs of an engulfed (top row) and an unengulfed (middle row) germ cell corpse in *chc-1(RNAi)* animals. Traces of membranes are shown in the left panels and boxed regions in the middle panels are magnified and shown in the right panels. Black arrows indicate gonadal sheath cell membranes. Black and white arrowheads indicate cell corpse membranes and germline syncytium membranes, respectively. Total numbers of germ cell corpses analyzed for each RNAi treatment are shown in the table (bottom row). * Data cited from our previous work [22].

To explore how the phagocytic receptor CED-1 and its downstream adaptor CED-6 function to induce cytoskeletal reorganization for cell corpse engulfment, we firstly sought to identify proteins that are in complex with endogenous CED-1 and/or CED-6. Using antibodies specific for the C-terminus of CED-1 (CED-1C) [22] and CED-6, we performed immunoprecipitations in whole cell lysates of wild type (N2), *ced-1(e1735)* and *ced-6(n1813)* strong loss-of-function mutants, and analyzed proteins in the precipitates using liquid chromatography-coupled tandem mass spectrometry (LC-MS/MS). Interestingly, multiple peptides of the heavy chain of clathrin (CHC-1) were identified from proteins co-precipitated with CED-1 and CED-6 in the N2 lysate but not in lysates of *ced-1(e1735)* or *ced-6(n1813)* mutants (Figure S1A and S1B). We therefore used RNA interference (RNAi) to deplete *chc-1* and examined the persistence of cell corpses in *C. elegans* germ lines. We found that germ cell corpses accumulated significantly in an age-dependent manner in *chc-1(RNAi)* animals. A similar increase was observed at 25°C in a *chc-1* temperature-sensitive mutant, *b1025ts* [35], though to a lesser extent than in *chc-1(RNAi)* animals (Figure 1A). These results indicate that loss of clathrin function caused accumulation of apoptotic cells in *C. elegans* germ lines. Previously it was also reported that clathrin RNAi induced an elevation in the number of germ cell corpses [23], but how clathrin functions in this process remains unclear.

Given the central role of clathrin in CME, we went on to investigate whether inactivation of other regulators required for CME could also result in accumulation of apoptotic cells. We used RNAi to deplete *C. elegans* homologs of individual mammalian proteins involved in CME and examined the persistence of germ cell corpses. Compared to animals with control RNAi, a significant increase in germ cell corpses was observed in animals treated with RNAi of *apa-2*, *apb-1* and *dpy-23*, which encode the α, β2 and μ2 subunits of the AP2 complex, respectively (Table S1). A time-course analysis confirmed that germ cell corpses increased significantly in an age-dependent manner in animals with RNAi of *apa-2*, *apb-1*, and *dpy-23*, but not *aps-2*, which encodes the σ2 subunit of the AP2 complex (Figure 1B). In addition, RNAi of *lst-4* and *dyn-1*, which encode *C. elegans* homologs of mammalian sorting nexins 9/18/33 and dynamin, respectively, also led to a strong accumulation of germ cell corpses (Table S1) [18], [22], [36]. Clathrin and the AP2 complex are essential for formation of CCVs while sorting nexin 9 and dynamin are required for scission of CCVs from the plasma membrane during endocytosis [32], [37].

To distinguish whether the increase in germ cell corpses in above RNAi animals resulted from excessive apoptosis or defective cell corpse clearance, we measured the duration of cell corpses using time-lapse recording. In animals with control RNAi, the average duration of germ cell corpses was 29.3±2.5 min (mean±SEM, standard error of the mean). In contrast, the majority of germ cell corpses in *chc-1(RNAi)* and *apb-1(RNAi)* animals persisted longer than 120 min, and no cell corpses existed less than 60 min (Figure 1C), suggesting that inactivation of clathrin or AP2 likely caused defective cell corpse clearance. To prove this, we performed transmission electronic microscopy (TEM) analysis to examine the engulfment of germ cell corpses. In *chc-1(RNAi)* animals, 28 out of 50 germ cell corpses examined (56%) from 5 gonad arms appeared not to be fully engulfed by sheath cells (Figure 1D). Similarly, 12 of 25 corpses (48%) from 7 gonad arms of *apb-1(RNAi)* animals were found not to be internalized (Figure 1D). In contrast, in animals treated with *gla-3* RNAi, which induces excessive germ cell apoptosis without affecting cell corpse clearance, 100% of cell corpses were fully internalized by gonad sheath cells (Figure 1D) [22], [38]. These findings indicate that the engulfment of cell corpses was impaired when clathrin and the AP2 complex were down-regulated.

### Clathrin and AP2 associate with phagosomes and act in the *ced-1* engulfment pathway

**Figure 2:**
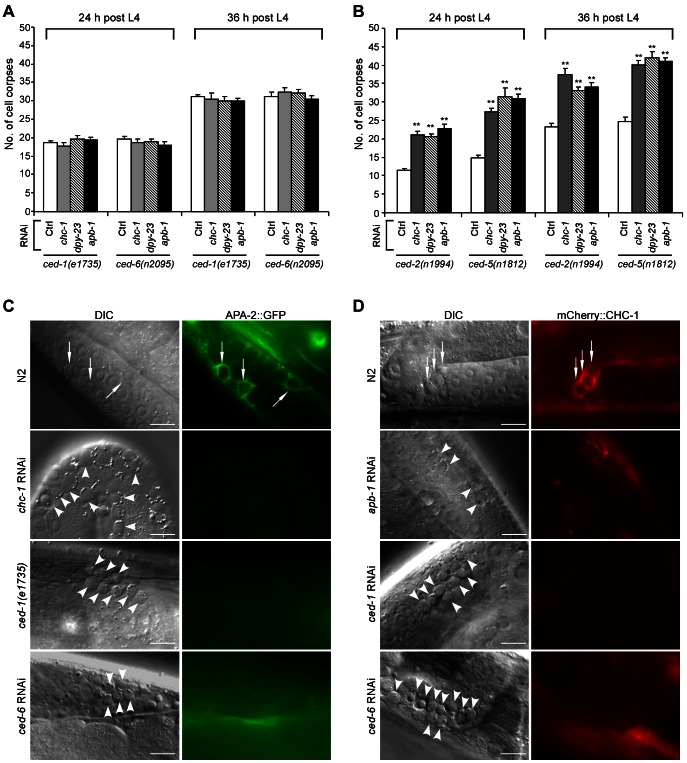
Clathrin and AP2 likely act in the same genetic pathway as CED-1 and CED-6. (A and B) Quantification of germ cell corpses in *ced-1(e1735)* and *ced-6(n2095)* (A) or *ced-2(n1994)* and *ced-5(n1812)* (B) mutants treated with Ctrl RNAi and RNAi of *chc-1*, *dpy-23* and *apb-1*. Cell corpses were scored in animals at 24 and 36 h after the L4 molt. Error bars represent SEM. Comparisons were made between control RNAi and RNAi of *chc-1*, *dpy-23* and *apb-1* using unpaired *t*-tests. ** *p*<0.001; all other points had *p*>0.05. (C) Representative images of cell corpse labeling by APA-2::GFP in N2, *ced-1(e1735)*, *chc-1(RNAi)* and *ced-6(RNAi*) germ lines. (D) Representative images of cell corpse labeling by mCherry::CHC-1 in N2, *apb-1(RNAi), ced-1(RNAi)* and *ced-6(RNAi)* germ lines. In (C) and (D) arrows indicate cell corpses labeled by APA-2::GFP or mCherry::CHC-1 while arrowheads indicate unstained corpses. Bars, 10 µm.

In *C. elegans*, the *ced-1*/*6*/*7* and *ced-2*/*5*/*12*/*10* signaling pathways act redundantly to mediate cell corpse engulfment [3]. As our findings revealed that CHC-1 and AP2 are involved in cell corpse engulfment, we tested whether depletion of individual AP2 components and *chc-1* could exert an additive effect on defective corpse engulfment in mutants deficient in either engulfment pathway. In *ced-1(e1735)* and *ced-6(n2095)* strong loss-of-function mutants in the cell corpse recognition pathway, RNAi of *apb-1*, *dpy-23*, or *chc-1* did not obviously change the numbers of germ cell corpses at all adult ages examined (Figure 2A). In contrast, RNAi depletion of these three genes significantly enhanced the numbers of germ cell corpses in *ced-2(n1994)* and *ced-5(n1812)* strong loss-of-function mutants affecting the cytoskeletal reorganization pathway (Figure 2B). These results suggest that *chc-1* and genes of the AP2 complex likely act within the same genetic pathway as *ced-1* and *ced-6* to regulate cell corpse clearance.

To determine whether clathrin and AP2 are directly involved in formation of cell corpse-containing phagosomes, we generated transgenes expressing green fluorescence protein (GFP)-tagged CHC-1, APA-2 and DPY-23 under the control of their own promoters. We found that these GFP-tagged proteins associated with phagosomes in the germ line (Figure S2A). Similarly, GFP-tagged APA-2 (APA-2::GFP) and mCherry-fused CHC-1 (mCherry::CHC-1) driven by the engulfing cell-specific *ced-1* promoter also appeared on phagosomes (Figure 2C and 2D). The fusions of CHC-1 with GFP and mCherry were functional in that both GFP::CHC-1 driven by the *chc-1* promoter (*yqEx480*) and mCherry::CHC-1 driven by the *ced-1* promoter (*yqIs98*) rescued the cell corpse phenotype in *chc-1(b1025ts)* mutants (Figure S2B). Likewise, APA-2::GFP driven by the *apa-2* promoter (*yqEx481*) or the *ced-1* promoter (*yqIs99*) rescued the accumulation of germ cell corpses in animals treated with RNAi complementary to the 3′ untranslated region (3′ UTR) of *apa-2* (Figure S2C). Interestingly, the labeling of cell corpses by APA-2::GFP was strongly inhibited by *chc-1* RNAi, and RNAi of *apa-2* and *apb-1* conversely suppressed corpse labeling by mCherry::CHC-1 (Figure 2C and 2D; Figure S7G; and data not shown). Thus, AP2 and CHC-1 likely form a complex and are recruited to phagosomes in a mutually dependent manner. Alternatively, CHC-1 is required for the stabilization of AP2 at the phagosome. Furthermore, in *ced-1(e1735), ced-1(RNAi)* and *ced-6(RNAi)* animals, few germ cell corpses were labeled by APA-2::GFP or mCherry::CHC-1, suggesting that CED-1 and CED-6 are required for phagosomal association of AP2 and clathrin (Figure 2C and 2D). On the other hand, we also examined the effect of loss of *chc-1* and *apb-1* on cell corpse recognition by CED-1::GFP and phagosomal recruitment of GFP::CED-6, both of which functionally rescued the defective cell corpse clearance in *ced-1(e1735)* and *ced-6(n2095)* mutants (Figure S2D and S2E). The surrounding of germ cell corpses by CED-1::GFP or GFP::CED-6 in *apb-1(RNAi)* and *chc-1(RNAi)* animals was similar to that in wild type and in *lst-4(tm2423)* mutants affecting phagosome maturation (see below) (Figure S3), indicating that loss of *apb-1* and *chc-1* did not affect cell corpse recognition by CED-1 and phagosomal recruitment of CED-6. Collectively, these data suggest that phagosomal recruitment of AP2 and clathrin occurs downstream of CED-1 and CED-6.

### CHC-1 and AP2 are required for actin rearrangement during phagocytosis

**Figure 3:**
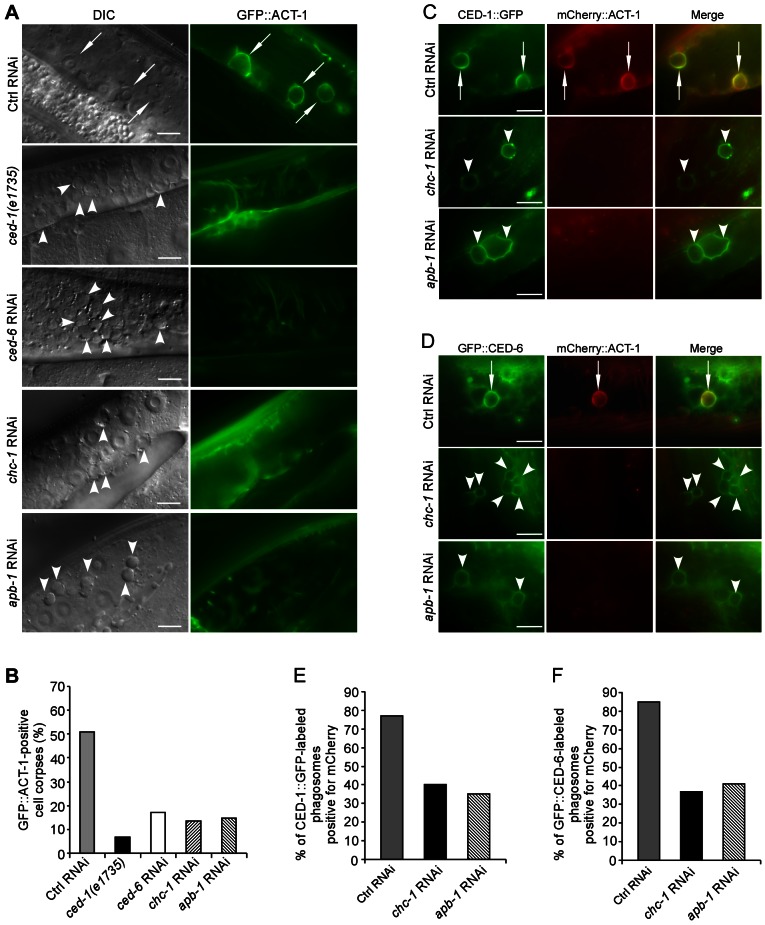
CHC-1 and AP2 are required for the rearrangement of the actin cytoskeleton necessary for cell corpse engulfment. (A) Representative images of cell corpse labeling by GFP::ACT-1 in *Ctrl(RNAi)*, *ced-1(e1735)*, *ced-6(RNAi)*, *chc-1(RNAi)* and *apb-1(RNAi)* germ lines. Arrows indicate cell corpses encircled by GFP-ACT-1 and arrowheads indicate unlabelled corpses. Bars, 10 µm. (B) Quantification of the labeling of germ cell corpses by GFP::ACT-1 as shown in (A). (C and D) Representative images of co-localization of mCherry::ACT-1 with CED-1::GFP (C) and GFP::CED-6 (D) on phagosomes. Arrows indicate phagosomes stained by both mCherry::ACT-1 and CED-1::GFP or GFP::CED-6 and arrowheads indicate phagosomes only positive for CED-1::GFP or GFP::CED-6. Bars, 10 µm. (E and F) Quantification of mCherry::ACT-1 labeling of CED-1::GFP-positive (E) and GFP::CED-6-positive phagosomes (F). In B, E and F, ≥100 corpses were scored for each genotype.

Next we investigated whether loss of clathrin or AP2 function affects the rearrangement of the actin cytoskeleton, which is required for internalization of cell corpses. For this purpose, we generated transgenes expressing GFP-fused ACT-1, an actin isoform that controls cytoplasmic microfilament function, and GFP-tagged *Drosophila* Moesin (GFP::Moesin) [39], a filamentous actin (F-actin)-specific-binding protein, both of which were driven by the engulfing cell-specific *ced-1* promoter. In wild-type animals, about 50% of germ cell corpses were surrounded by GFP::ACT-1. In *ced-1(e1735)* or *ced-6(RNAi)* animals, however, the labeling of germ cell corpses by GFP::ACT-1 was strongly reduced (Figure 3A and 3B). Similar reduction in labeling of germ cell corpses by GFP::ACT-1 was observed in *chc-1(RNAi)* and *apb-1(RNAi)* animals (Figure 3A and 3B). Similarly, whereas about 60% of germ cell corpses were positive for GFP::Moesin in the wild type, only 10–20% of germ cell corpses were encircled by GFP::Moesin in animals treated with RNAi of *ced-1*, *chc-1* or *apb-1* (Figure S4). Thus, loss of *chc-1* and *apb-1* resulted in a failure in actin cytoskeleton rearrangement required for cell corpse engulfment, like that caused by loss of *ced-1* or *ced-6*. To prove this, we examined the recruitment of mCherry-tagged ACT-1 by phagosomes positive for CED-1::GFP or GFP::CED-6 in *chc-1(RNAi)* and *apb-1(RNAi)* animals. RNAi of *chc-1* or *apb-1* caused a strong decrease in labeling of CED-1::GFP-positive phagosomes by mCherry::ACT-1 from 77% to 35–40% (Figure 3C and 3E). Similarly, only 37–41% of GFP-CED-6-positive phagosomes were labeled by mCherry::ACT-1 in animals with RNAi of *chc-1* or *apb-1*, compared to 85% in animals with control RNAi (Figure 3D and 3F). Taken together, these findings suggest that CHC-1 and AP2 act downstream of CED-1 and CED-6 to mediate the rearrangement of the actin cytoskeleton required for corpse engulfment.

### CHC-1 and AP2 form a complex with CED-1 and CED-6

**Figure 4:**
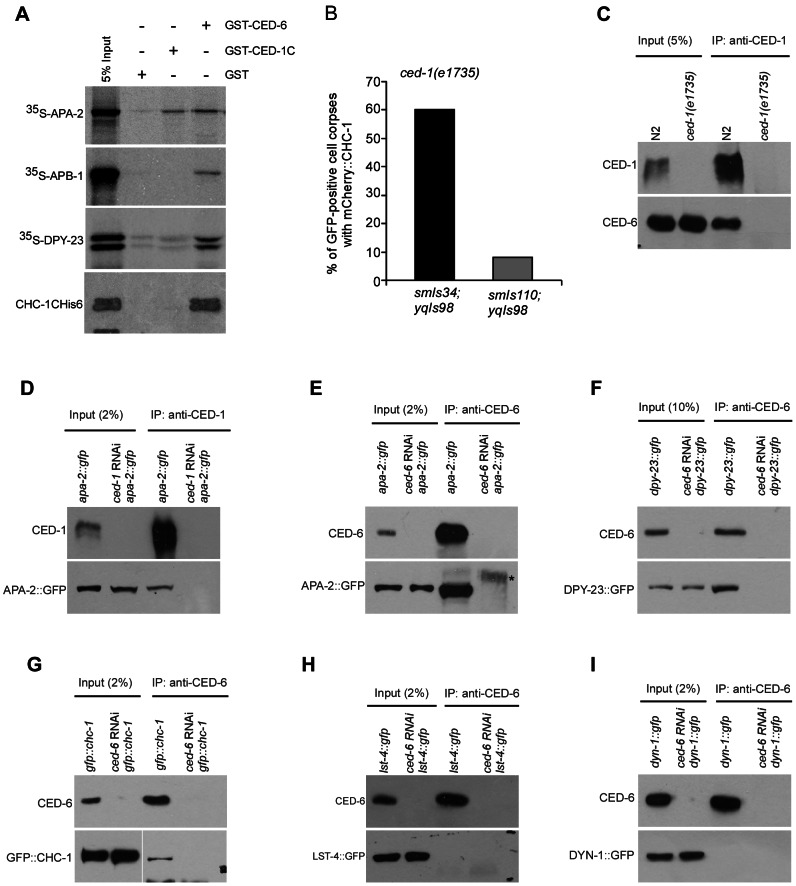
CHC-1 and AP2 components interact with CED-1 or CED-6. (A) ^35^S-labeled APA-2, APB-1, DPY-23 and His6-tagged CHC-1C (amino acids 825–1682) were incubated with immobilized GST, GST-CED-1C (amino acids 933–1111) and GST-CED-6. After extensive washing, bound proteins were viewed by autoradiography or detected using immunoblotting with His6 antibody. (B) Quantification of mCherry::CHC-1(*yqIs98*) labeling of CED-1::GFP (*smIs34*)- or CED-1ΔC::GFP (*smIs110*)-positive cell corpses in *ced-1(e1735)* adult germ lines. ≥50 GFP-positive cell corpses were scored from germ lines 48 h post the L4 molt. (C) CED-6 associated with CED-1 immunoprecipitated from N2 but not *ced-1(e1735)* cell lysates. CED-1C antibody was used for immunoprecipitations (IPs) and precipitated proteins were detected with CED-1C and CED-6 antibodies. (D and E) APA-2::GFP associated with CED-1 (D) and CED-6 (E) immunoprecipitated from lysates of APA-2::GFP-expressing animals but not the same animals treated with RNAi of *ced-1* or *ced-6*. CED-1C or CED-6 antibodies were used for IPs and precipitates were detected using antibodies against GFP, CED-1C and CED-6. The asterisk indicates a non-specific band. (F and G) DPY-23::GFP and GFP::CHC-1 associated with CED-6 immunopreciptated from lysates of animals expressing DPY-23::GFP (F) or GFP::CHC-1 (G) but not the same animals treated with *ced-6* RNAi. CED-6 antibody was used for IP and precipitates were detected with CED-6 and GFP antibodies. (H and I) LST-4::GFP and DYN-1::GFP did not associate with CED-6 immunoprecipitated from lysates of animals expressing LST-4::GFP (H) or DYN-1::GFP (I). CED-6 antibody was used for IP and precipitated proteins were detected with CED-6 and GFP antibodies.

To understand how clathrin and AP2 may cooperate with CED-1 and CED-6 to control cytoskeletal rearrangement in the CED-1 cell corpse engulfment pathway, we first tested whether AP2 components and CHC-1 can physically interact with CED-1 using in vitro GST pull-down assays. The GST-fused intracellular region of CED-1 (CED-1C, amino acids 933–1111), but not GST, directly interacted with ^35^S-labeled APA-2 synthesized by in vitro translation (Figure 4A). However, other AP2 subunits labeled by ^35^S and the purified recombinant C-terminal region of CHC-1 (CHC-1C, amino acids 825–1628) did not show a detectable interaction with CED-1C, suggesting that CED-1 likely interacts with the AP2 complex through its α subunit (Figure 4A). To determine whether the intracellular region of CED-1 is important for phagosomal recruitment of CHC-1 and/or AP2, we examined if expression of a GFP-fused CED-1 with the C-terminal region deleted (CED-1ΔC::GFP, *smIs110*) could rescue the defective phagosomal recruitment of mCherry::CHC-1 in *ced-1(e1735)* mutants. CED-1ΔC::GFP failed to rescue the cell corpse phenotype (Figure S2D) but labeled cell corpses in *ced-1(e1735)* mutants; however, barely any mCherry::CHC-1 was found to co-localize with CED-1ΔC::GFP (Figure 4B). In contrast, the full-length CED-1::GFP (*smIs34*) fully rescued the cell corpse phenotype in *ced-1(e1735)* animals (Figure S2D) and about 60% of CED-1::GFP-positive cell corpses were labeled by mCherry::CHC-1. Thus the intracellular region of CED-1 is required for phagosomal recruitment of CHC-1.

We also tested the interaction of GST-fused CED-6 with individual AP2 components and CHC-1, and found that GST-CED-6 directly interacted with ^35^S-labeled APA-2, APB-1 and DPY-23, and His6-tagged CHC-1C (Figure 4A). These findings suggest the possibility that CED-1 and CED-6 form a complex with the AP2 complex and CHC-1. To prove this, we examined the interaction of CHC-1 and individual components of the AP2 complex with endogenous CED-1 and CED-6 by performing immunoprecipitations with CED-1C- or CED-6-specific antibodies. We found that endogenous CED-6 associated with CED-1 immunoprecipitated by the CED-1C antibody, providing direct evidence that CED-1 and CED-6 form a complex in *C. elegans* (Figure 4C). Importantly, APA-2::GFP was co-immunoprecipitated with endogenous CED-1 and CED-6 (Figure 4D and 4E), and similar co-immunoprecipitation of DPY-23::GFP and GFP::CHC-1 with endogenous CED-6 was observed (Figure 4F and 4G). The specificity of these in vivo protein interactions was supported by the absence of any interaction, using the same immunoprecipitation assay, between CED-6 and GFP-fused LST-4 and DYN-1, two factors required for phagosome maturation (see below) (Figure 4H and 4I). Thus the in vivo interactions of CHC-1 and individual AP2 components with CED-6 or CED-1 are consistent with their direct interactions in vitro, suggesting that the AP2 complex and CHC-1 likely fulfill their functions in cell corpse engulfment by forming a complex with CED-1 and CED-6.

### Loss of *chc-1* and AP2 function block phagosome maturation

**Figure 5:**
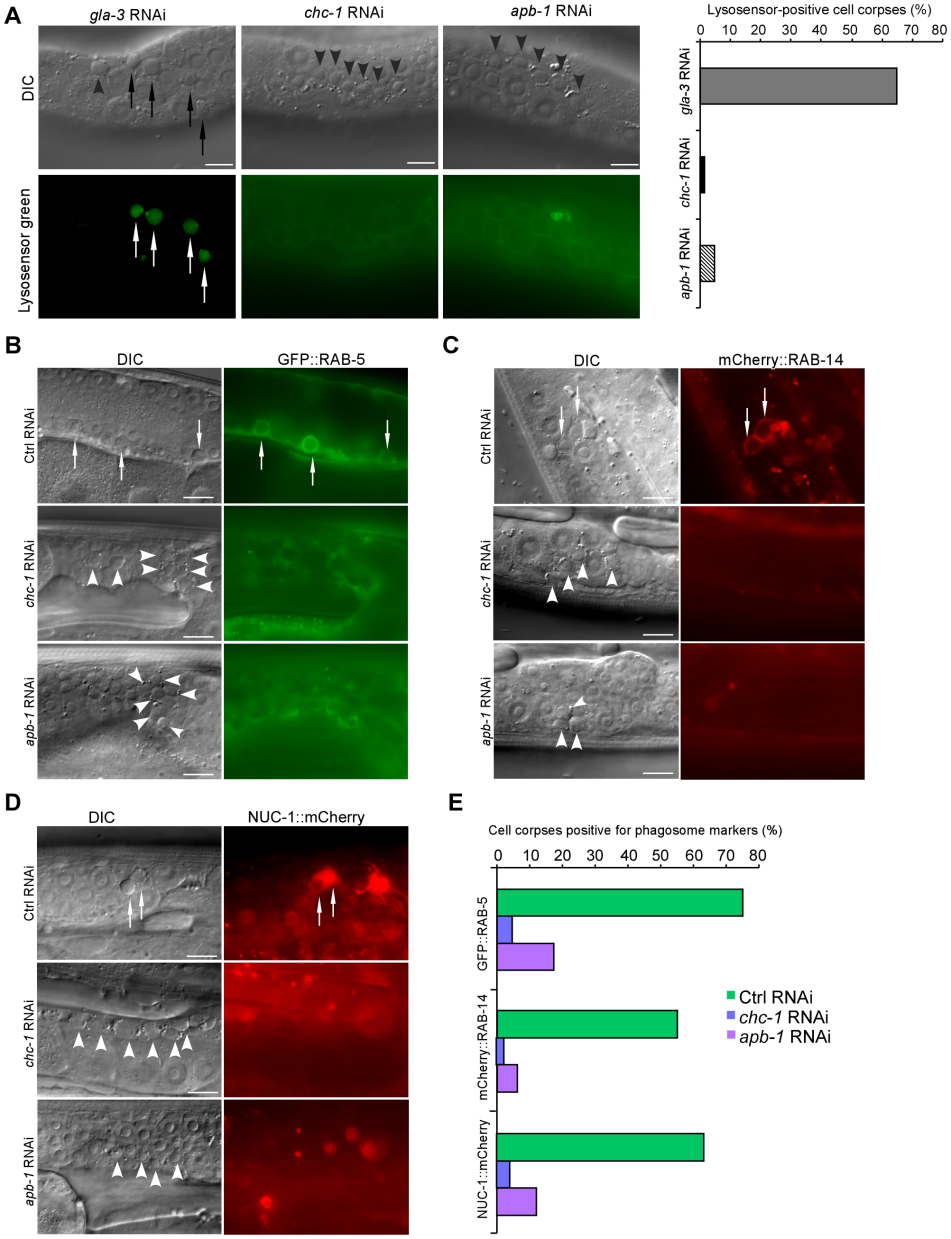
CHC-1 and AP2 are required for phagosome maturation. (A) Representative images of germ cell corpse staining by LysoSensor Green DND-189 in *gla-3(RNAi), chc-1(RNAi)* and *apb-1(RNAi)* animals. Quantification of corpse staining is shown on the right. ≥100 corpses were analyzed for each RNAi treatment. (B–D) Representative DIC and fluorescence images of germ cell corpse labeling by GFP::RAB-5 (B), mCherry::RAB-14 (C), and NUC-1::mCherry (D) in *Ctrl(RNAi)*, *apb-1(RNAi)*, and *chc-1(RNAi)* animals. (E) Quantification of cell corpse labeling as shown in (B–D). ≥100 corpses were analyzed for each genotype. In (A–D), arrows indicate cell corpses labeled by phagosomal markers; arrowheads indicate unlabeled corpses. Bars, 10 µm.

As EM analysis indicated that a significant proportion of germ cell corpses were still internalized by sheath cells in *chc-1(RNAi)* and *apb-1(RNAi)* animals, we wondered whether maturation of phagosomes containing cell corpses was affected in these animals. To assess this, we examined the acidification of phagosomes with LysoSensor Green DND-189, an indicator of normal progression of phagosome maturation. We found that germ cell corpses were mostly negative for LysoSensor Green DND-189 staining in *chc-1(RNAi)* and *apb-1(RNAi)* animals compared to animals with *gla-3* RNAi that induces an elevation in apoptosis without affecting cell corpse clearance [38], suggesting that the maturation of phagosomes was inhibited (Figure 5A). To corroborate this conclusion, we examined phagosomal recruitment of several effectors essential for phagosome maturation in *apb-1(RNAi)* and *chc-1(RNAi)* germ lines, including GFP-fused RAB-5 (GFP::RAB-5) and mCherry-fused RAB-14 (mCherry::RAB-14), two small GTPases required for phagosomal progression from early to late stages, and mCherry-fused NUC-1 (NUC-1::mCherry), a lysosomal DNase that indicates the formation of phagolysosomes [27]. We found that the labeling of cell corpses by these phagosomal markers in *apb-1(RNAi)* and *chc-1(RNAi)* animals was greatly reduced compared to that in wild type (Figure 5B–5E), indicating that phagosomes in these animals arrested at an early stage of maturation. Taken together, these data indicate that clathrin and AP2 act at an early stage of phagosome maturation, impairment of which inhibited the progression of phagosomes from early to late stages.

### LST-4 acts at an early stage of phagosome maturation

To elucidate how AP2 and CHC-1 function in phagosome maturation in addition to their role in cell corpse engulfment, we sought to determine their functional interactions with two other regulators identified in our screen, LST-4 and DYN-1, the *C. elegans* homologs of mammalian Snx9/18/33 and dynamin, respectively. Snx9 and dynamin act together with AP2 and clathrin to regulate the formation of CCVs in CME [32], [40], [41]. DYN-1 was previously shown to act at an early stage of phagosome maturation by forming a complex with VPS-34 and RAB-5 whereas LST-4 likely affects cell corpse degradation at a similar stage to DYN-1 [22], [23], [36], [42]. As the first step towards our goal, we set out to clarify the role of LST-4 in phagosome maturation by comparing the cell corpse phenotype of two deletion mutants, *tm2423* and *qx159*. We found that these mutants accumulate germ cell corpses to similar levels in an age-dependent manner (Figure S5A and S5B). In addition, around 70% of *lst-4(tm2423)* germ cell corpses were found to be encircled by GFP::Moesin, compared with 60% in wild type, indicating that loss of *lst-4* did not affect cell corpse internalization (Figure S5C and S5H). However, germ cell corpses labeled by the early phagosome markers YFP::2xFYVE, GFP::RAB-5, and mCherry::RAB-14, and the late phagosome marker GFP::RAB-7, were greatly reduced in *lst-4(tm2423)* animals, indicating that loss of *lst-4* inhibited the recruitment of factors required for phagosome maturation (Figure S5D–S5H). Moreover, loss of *lst-4* also blocked phagosome acidification as the majority of germ cell corpses were negative for LysoSensor Green DND-189 staining in either *lst-4(tm2423)* single mutants or double mutants of *lst-4(tm2423)* with *vps-18(tm1125)* that was previously shown to cause defective phagolysosome formation but not phagosome acidification [29] (Figure S6A and S6B). This contrasts to the high proportion of corpses stained by the same dye in *gla-3(RNAi)* animals, in which cell corpses are normally removed, and in *vps-18(tm1125)* single mutants (Figure S6A and S6B). Importantly, we found that LST-4 was recruited to phagosomes using LST-4::GFP or LST-4::mCherry fusions that fully rescued the cell corpse phenotype in *lst-4(tm2423)* mutants, even though they appeared cytoplasmic in several tissues (Figure S6C–S6E; Figure S7A). The phagosomal association of LST-4 was blocked by loss of *ced-1* and *ced-6* but not RNAi depletion of *dyn-1*, *rab-5* and *rab-7*, three genes required for phagosome maturation but not corpse engulfment (Figure S6E and S6F). Together, these findings, in agreement with the results obtained by Almendinger et al. [36], establish that LST-4 acts at an early stage of phagosome maturation.

### LST-4 functions through DYN-1 to promote phagosome maturation

**Figure 6:**
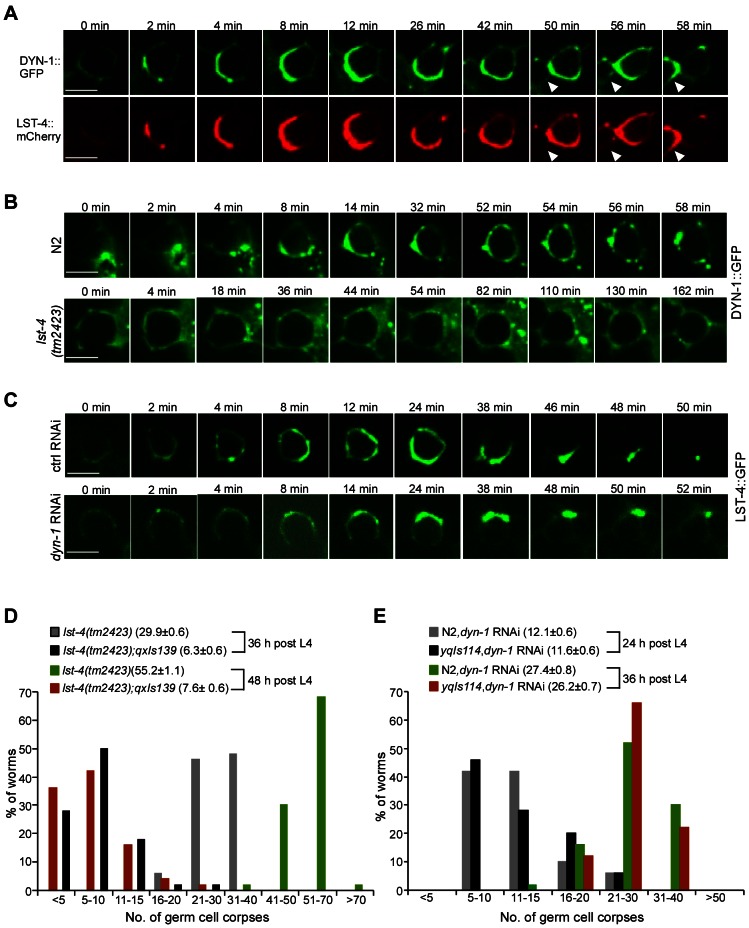
LST-4 acts through DYN-1. (A) Representative time-lapse images of phagosomal association of DYN-1::GFP and LST-4::mCherry. Images were taken with a spinning disk confocal microscope. The time point when DYN-1 formed a weak ring was defined as 0 min. Arrowheads indicate a newly formed cell corpse. Bars, 5 µm. (B) Representative time-lapse images of phagosomal association of DYN-1::GFP in N2 and *lst-4(tm2423)* animals. Images were taken and analyzed as in (**A**). Bars, 5 µm. (C) Representative time-lapse images of germ corpses in *yqIs114* (P*_lst-4_lst-4*(cDNA)::*gfp*) animals treated with Ctrl RNAi and *dyn-1* RNAi. Adult animals (24 h after the L4 molt) were observed and images were taken and analyzed as above. Bars, 5 µm. In (A–C), ≥15 germ cell corpses were recorded. (D) Quantification of germ cell corpses in *lst-4(tm2423)* and *lst-4(tm2423);qxIs139* (P*_ced-1_dyn-1*::*gfp*) animals at 36 and 48 h after the L4 molt. A total of 50 gonad arms from 50 animals were examined for each strain at every time point. The *x*-axis represents the number of cell corpses and the *y*-axis represents the % of animals. The average number of germ cell corpses per gonad arm (mean±SEM) is shown in parenthesis. (E) Quantification of germ cell corpses in N2 and *yqIs114* (P*_lst-4_lst-4*(cDNA)::*gfp*) animals treated with *dyn-1* RNAi at 24 and 36 h after the L4 molt, respectively. Cell corpses were scored and analyzed as in (D).

We next characterized the functional interaction between LST-4 and DYN-1. In animals co-expressing LST-4::mCherry and DYN-1::GFP, which is able to rescue the defective cell removal in *dyn-1(ky51)* mutants, both proteins were found to colocalize on phagosomes (Figure S7A and S7B). Time-lapse analysis revealed that both proteins were simultaneously recruited to the phagosome and quickly formed a crescent-like structure, before dissociating from the phagosome at the same time (Figure 6A). Using immunoprecipitation we further found that these two proteins associated with one another in *C. elegans* (Figure S7C, top panel) whereas they did not show detectable in vivo interaction with CED-6 (Figure 4H and 4I). Consistent with this, His6-tagged recombinant LST-4 directly interacted with GST-fused DYN-1, which confirmed the in vitro interaction of these two proteins reported previously [42]. Nevertheless, no interaction of LST-4His6 or DYN-1His6 with CED-1C or CED-6 was detected in the same GST pull-down assay (Figure S7C, bottom panel). Together these results indicate that LST-4 and DYN-1 form a complex to regulate phagosome maturation but do not act in complex with CED-1 or CED-6. To further determine the effect of LST-4-DYN-1 interaction on their association with phagosomes, we monitored the dynamic association of DYN-1::GFP with phagosomes in germ lines of wild-type and *lst-4(tm2423)* animals, and phagosomal association of LST-4::GFP in wild-type and *dyn-1(RNAi)* germ lines. In the wild type, DYN-1 was initially localized to the periphery of the phagosome and then quickly became enriched to form a large patch-like structure (Figure 6B, 0–14 min). DYN-1 then became more evenly distributed on the phagosome before forming punctate structures (Figure 6B, 14–56 min), which likely represent the dissociation of DYN-1 from the phagosome. Unlike in wild-type, DYN-1::GFP neither became sharply enriched nor formed an obvious patch on phagosomes in *lst-4(tm2423)* mutant germ lines (Figure 6B), suggesting that loss of *lst-4* likely affected the enrichment or stabilization of DYN-1 on phagosomes. These findings are in agreement with the observations made previously by Lu et al. that loss of *lst-4* impaired the phagosomal association of DYN-1 during embryonic cell corpse removal [42]. In addition, we noticed that DYN-1::GFP was more enriched on phagosomes when co-expressed with LST-4::mCherry (compare Figure 6A and 6B). On the other hand, RNAi depletion of *dyn-1* seemed not to affect the association of LST-4 with phagosomes, because no obvious difference in the dynamic association of LST-4::GFP with phagosomes was observed between *dyn-1(RNAi)* and control RNAi animals (Figure 6C). These results, together with the findings made by Lu et al. and Almendinger et al. [36], [42], establish that LST-4 promotes phagosomal activity of DYN-1. Importantly, we further found that *lst-4(tm2423)* mutants expressing DYN-1::GFP (*qxIs139*) displayed an obvious reduction in germ cell corpses compared with the same mutants without DYN-1::GFP expression (Figure 6D). For example, *lst-4(tm2423)* animals expressing DYN-1::GFP (*qxIs139*) contained 6.3±0.6 (mean±SEM) and 7.6±0.6 corpses per gonad arm at adult ages of 36 and 48 h post L4, respectively, compared with 29.9±0.6 and 55.2±1.1 in *lst-4(tm2423)* mutants (Figure 6D). In contrast, *dyn-1* RNAi caused similar levels of germ cell corpse accumulation in both wild type and animals expressing LST-4::GFP (*yqIs114*) (Figure 6E). Taken together, these findings provide strong evidence that LST-4 forms a complex with DYN-1 and acts through the latter to promote the initiation of phagosome maturation.

### Clathrin and AP2 form a complex with LST-4 and DYN-1 in phagosome maturation

**Figure 7:**
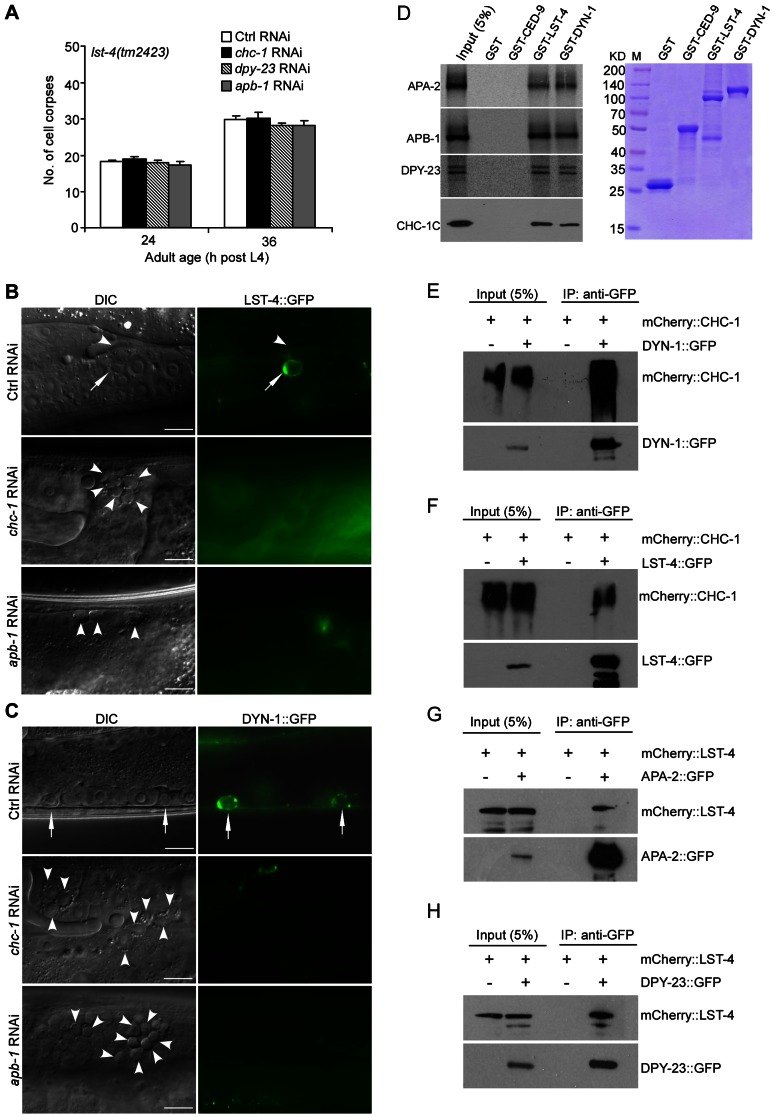
Clathrin and AP-2 function upstream of LST-4 and DYN-1 for apoptotic cell clearance. (A) Quantification of germ cell corpses in *lst-4(tm2423)* mutants treated with Ctrl RNAi and RNAi of *chc-1*, *dpy-23* and *apb-1*. Error bars represent SEM. Comparisons were performed between Ctrl RNAi and RNAi of *chc-1*, *dpy-23* and *apb-1* using unpaired *t*-tests. All points had *p*>0.05. (B and C) Representative images of phagosomal association of LST-4::GFP (B) and DYN-1::GFP (C) in *Ctrl(RNAi)*, *chc-1(RNAi)* and *apb-1(RNAi)* animals. Arrows point to germ cell corpses labeled by LST-4::GFP or DYN-1::GFP and arrowheads indicate unlabeled corpses. Bars, 10 µm. (D) On the left, ^35^S-labeled APA-2, APB-1, DPY-23 and His6-tagged CHC-1C were incubated with immobilized GST, GST-CED-9, GST-LST-4 and GST-DYN-1 (3 ug of each). Bound proteins were resolved on sodium dodecyl sulfate polyacrylamide gels and viewed by autoradiography or detected by immunoblotting with His6 antibody. GST and GST-fused proteins used for binding are shown in the right panel. (E and F) mCherry-tagged CHC-1 associated with DYN-1::GFP (E) and LST-4::GFP (F) in animals. IPs were performed with GFP antibody on lysates of animals expressing mCherry::CHC-1 (*yqIs98*) alone, animals co-expressing mCherry::CHC-1 and DYN-1::GFP (*yqIs98;qxIs139*), and animals co-expressing mCherry::CHC-1 and LST-4::GFP (*yqIs98;yqIs114*). Precipitates were detected by immunoblotting with antibodies for mCherry and GFP, respectively. (G and H) mCherry-tagged LST-4 associated with APA-2::GFP (G) and DPY-23 (H) in animals. IPs were performed with GFP antibody on lysates of animals expressing mCherry::LST-4 alone (*yqIs119*), animals co-expressing mCherry::LST-4 and APA-2::GFP (*yqIs119;yqIs99*), and animals co-expressing mCherry::LST-4 and DPY-23::GFP *(yqIs119;yqIs120)*. Precipitates were detected by immunoblotting with mCherry and GFP antibodies.

Having demonstrated that both CHC-1-AP2 and LST-4-DYN-1 complexes act at a very early stage of phagosome maturation, we asked how CHC-1 and AP2 might affect LST-4 and DYN-1. Firstly, we tested if depletion of *chc-1* and AP2 had an additive role in cell corpse accumulation in *lst-4(tm2423)* animals, and found that RNAi of *chc-1*, *apb-1*, and *dpy-23* did not affect the numbers of germ cell corpses in *lst-4(tm2423)* mutants (Figure 7A). Secondly, we investigated whether phagosomal association of LST-4 and DYN-1 were affected by inactivating *chc-1* and the AP2 complex. In *chc-1(RNAi)* and *apb-1(RNAi)* germ lines, the labeling of germ cell corpses by LST-4::GFP and DYN-1::GFP was strongly reduced compared to that in wild type (Figure 7B and 7C; Figure S7D), indicating that CHC-1 and AP2 are important for phagosomal association of LST-4 and DYN-1. In contrast, phagosomal association of APA-2::GFP and mCherry::CHC-1 in *lst-4(tm2423)*, *dyn-1(ky51)*, or *dyn-1(RNAi)* animals were similar to that in wild type (Figure S7E–S7G). These results suggest that CHC-1 and the AP2 complex function upstream of LST-4 and DYN-1 in phagosome maturation. Finally, we tested whether CHC-1 and individual AP2 subunits could directly interact with LST-4 and/or DYN-1. As shown in Figure 7D, ^35^S-labeled APA-2, APB-1, and DPY-23 interacted with GST-fused DYN-1 and LST-4, but not GST or GST-fused CED-9, an anti-apoptotic protein acting at the cell-killing stage [43]. Similarly, purified recombinant CHC-1C interacted with GST-DYN-1 and GST-LST-4 but not GST or GST-fused CED-9. Thus clathrin and AP2 likely form a complex with LST-4 and DYN-1. To prove this, we examined whether DYN-1 and LST-4 are indeed in complex with CHC-1 and AP2 in *C. elegans*. Using immunoprecipitation we found that mCherry::CHC-1 associated with DYN-1::GFP in animals co-expressing these two proteins (Figure 7E). Similarly, mCherry::CHC-1 and LST-4::GFP associated with one another as revealed by co-immunoprecipitation (Figure 7F). In addition, we found that mCherry::LST-4 co-immunoprecipitated with APA-2::GFP and DPY-23::GFP (Figure 7G and 7H), indicating that LST-4 interacts with AP2 components in *C. elegans*. Taken together, the in vitro and in vivo protein interactions among LST-4, DYN-1, CHC-1 and AP2 components (Figure 7D–7H, Figure S7C) strongly suggest that clathrin and AP2 form a complex with LST-4 and DYN-1, thereby promoting phagosome maturation during cell corpse clearance.

## Discussion

**Figure 8:**
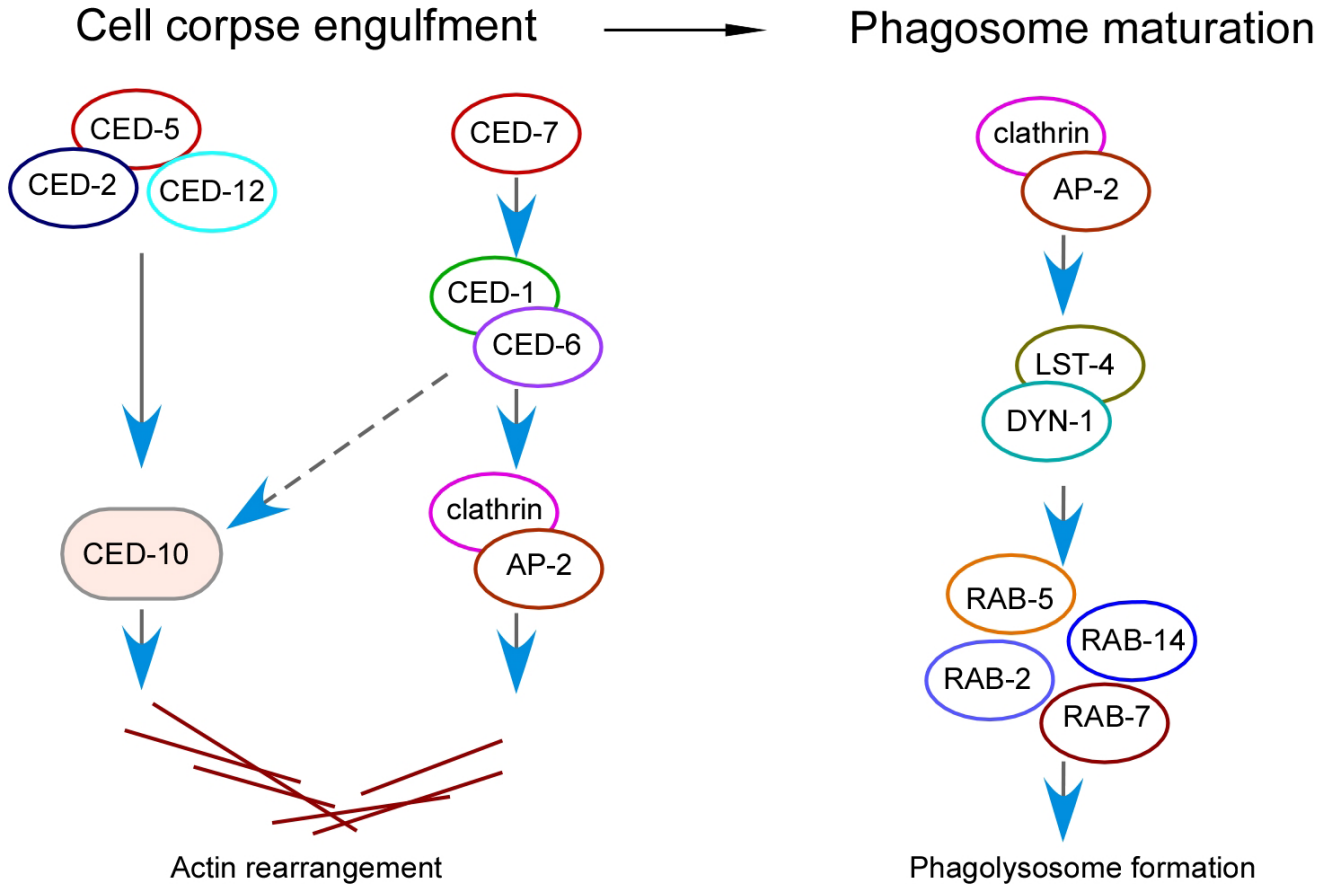
Schematic summary of the role of clathrin and the AP2 complex in both corpse engulfment and phagosome maturation during phagocytosis of apoptotic cells. In the cell corpse engulfment phase, clathrin and AP2 act downstream of CED-1 and CED-6 to promote actin rearrangement, which is required for phagocytosis. In the phagosome maturation phase, clathrin and AP2 promote phagosomal association of LST-4 and DYN-1, which initiates the maturation process.

During phagocytosis, the phagocytic receptor CED-1 recognizes cell corpses and transduces engulfment signals to the CED-6 adaptor. DYN-1/dynamin was also reported to participate in the *ced-1* pathway for corpse engulfment, and likely acts downstream of CED-1 and CED-6 [18]. Nevertheless, it is not clear how these factors coordinate to induce the rearrangement of the actin cytoskeleton, a key event required for cell corpse internalization. Although it was previously proposed that the two engulfment pathways for cytoskeletal reorganization converged on the CED-10 GTPase, the molecular link between the phagocytic receptor CED-1 and CED-10 remains to be identified [44]. In this study, we explored the role of major regulators of CME, a process that internalizes cell surface materials by use of clathrin-coated vesicles, in phagocytosis of apoptotic cells. Our findings revealed that clathrin and the AP2 complex are essential players in the process of cell corpse engulfment. Inactivation of the clathrin heavy chain CHC-1 or individual components of AP2 resulted in accumulation of cell corpses in the *C. elegans* germ line. Moreover, RNAi of *chc-1* or AP2 components significantly enhanced the engulfment defects in *ced-2* and *ced-5* strong loss-of-function mutants but not mutants deficient in *ced-1* and *ced-6*, suggesting that the *chc-1* and AP2 genes likely act within the same genetic pathway as *ced-1* and *ced-6*. Our results demonstrated that CHC-1 and the AP2 complex associate with phagosomes containing cell corpses in an inter-dependent manner and their phagosomal recruitment requires CED-1 and CED-6. Importantly, loss of clathrin or AP2 function severely impaired the rearrangement of the actin cytoskeleton required for corpse engulfment. Altogether these findings provide strong evidence that clathrin and AP2 function downstream of CED-1 and CED-6 and likely mediate the cytoskeletal reorganization required for cell corpse internalization (Figure 8).

Our findings suggest that clathrin and the AP2 complex serve a dual role in the process of apoptotic cell removal (Figure 8). On one hand, clathrin and AP2 are important for cell corpse engulfment by acting downstream of CED-1 and CED-6 to mediate cytoskeletal rearrangement in engulfing cells (Figure 8). This function is likely achieved by forming a protein complex with CED-1 and CED-6. In support of this conclusion, we found that CED-1 indeed forms a complex with CED-6 in vivo. Remarkably, our immunoprecipitation and in vitro GST pull-down results revealed that the CED-6 adaptor protein directly interacts with CHC-1 and individual components of the AP2 complex in *C. elegans*. Interestingly, we found that the CED-1 receptor likely interacts with AP2 via the α subunit of the latter. Because clathrin can function as an actin organizer at large membrane interfaces that far exceed the size of conventional CCVs [45] and loss of CHC-1 and AP2 function caused defective recruitment of actin around cell corpses, we propose that the formation of a protein complex by CED-1, CED-6, AP2 and CHC-1 provides a hub for recruitment and assembly of actin for cell corpse engulfment. On the other hand, clathrin and the AP2 complex are essential for phagosome maturation following corpse internalization. Loss of CHC-1 and AP2 function abrogated the acidification of phagosomes and inhibited phagosomal recruitment of downstream effectors required for phagosome maturation. In addition, our data demonstrated that LST-4 interacts with DYN-1 to promote its association with phagosomes. Clathrin and AP2 facilitate phagosomal association of the LST-4-DYN-1 complex by interacting with them, thereby promoting the initiation of phagosome maturation (Figure 8). Notably, whereas AP2 and CHC-1 were found to form complexes with either CED-1-CED-6 or LST-4-DYN-1, no protein interaction of CED-1 or CED-6 with LST-4 or DYN-1 was detected by either co-immunoprecipitation or GST pull-down assays. Thus clathrin and AP2 likely form two types of complex with factors required for engulfment and phagosome maturation, establishing them as a molecular link between engulfment and phagosome maturation in apoptotic cell clearance mediated by the phagocytic receptor CED-1 (Figure 8).

The recruitment of actin around germ cell corpses mediated by the complex of CED-1, CED-6, AP2 and clathrin may resemble the pathway used by mammalian cells to phagocytose pathogens. In mammalian cells, clathrin and some other regulators of CME are found to be essential for invasion of pathogenic bacteria, fungi and large viruses [34], [46]–[51]. For example, clathrin and dynamin were found to localize to bacterial entry foci during the invasion of *Listeria monocytogenes* and inactivation of major regulators of CME, such as Grb2, EPS15, CIN85 and CD2AP, severely inhibited bacterial internalization [34]. Further studies revealed that upon bacterial infection, the clathrin heavy chain CHC undergoes Src-dependent phosphorylation, which in turn initiates the accumulation of clathrin coats at bacterial adhesion sites. Through interaction of the clathrin light chain CLC with the actin-interacting protein Hip1R, actin is recruited and assembled at bacteria-host adhesion sites, leading to bacterial internalization. Thus the clathrin-coated pits that accumulate at bacterial entry sites serve as platforms for the actin polymerization needed for phagocytosis [51]. Intriguingly, the clathrin adaptor Dab2, but not AP2, is critical for clathrin recruitment to *L. monocytogenes* entry sites [34]. In *C. elegans*, our findings indicate that clathrin is similarly required for the actin rearrangement needed for phagocytosis of apoptotic cells. Nevertheless, RNAi depletion of *hipr-1* and *clic-1*, which encode *C. elegans* homologs of mammalian HipR1 and clathrin light chain, respectively, did not induce a similar level of corpse accumulation to that caused by *chc-1* RNAi (Table S1). We also performed RNAi to deplete several other putative actin-binding proteins predicted by the STRING protein interaction prediction program (http://string-db.org/) but failed to detect an obvious accumulation of germ cell corpses (data not shown). Thus it is possible that multiple factors may function redundantly to mediate the recruitment of actin by the CED-1-CED-6-AP2-clathrin complex. Further studies will be necessary to unveil the underlying mechanism. In addition, unlike clathrin recruitment during bacterial phagocytosis by mammalian cells, phagosomal recruitment of clathrin requires the AP2 complex in *C. elegans*. The requirement for different adaptors may be attributed to the use of different receptors for engulfment of apoptotic cells and bacteria. Besides, as the sizes of cell corpses in *C. elegans* are normally ≥1 µm, which is much larger than endocytic CCVs (<200 nm), it also remains to be determined how clathrin is assembled (i.e, clathrin *per se*, clathrin-coated vesicles, or clathrin-coated pits) when it associates with phagosomes. Moreover, the engulfment of cell corpses in *C. elegans* appears to involve fewer CME regulators compared with mammalian phagocytosis of pathogens. In our unbiased RNAi screen of *C. elegans* CME regulators, we found that only CHC-1 and AP2 components obviously affected cell corpse engulfment and degradation while LST-4/Snx9/18/33 and DYN-1/dynamin were essential for phagosome maturation; in contrast, RNAi inactivation of several major CME regulators has been shown to inhibit bacterial infection of mammalian cells. Thus, whereas both cell corpse engulfment in *C. elegans* and pathogen invasion in mammals make use of clathrin for actin rearrangement, other factors may differ owing to the requirement of distinct signaling mechanisms.

Remarkably, MEGF10, the mammalian ortholog of CED-1, was reported to interact with the μ2 subunit of AP2 in a yeast 2-hybrid screen, and the existence of a protein complex containing MEGF10 and AP2 subunits was further confirmed by a protein purification assay [52], [53]. More recently, *Drosophila* Ced-6 was identified as a clathrin-associated sorting protein (CLASP) as it binds to clathrin and AP2 via the C-terminal region [54]. Furthermore, the phosphotyrosine-binding domain (PTB domain) of *Drosophila* Ced-6 specifically recognizes a noncanonical sorting signal in the vitellogenin receptor Yolkless. Thus Ced-6 participates in clathrin-mediated yolk uptake in *Drosophila* egg chambers [54]. In addition, the mammalian homolog of CED-6, Gulp, can also interact with both clathrin and AP2 [54], [55]. In our study we found that clathrin and AP2 act in phagocytic receptor-mediated cell corpse removal by forming a protein interaction cascade with CED-1 and CED-6 to regulate the actin rearrangement required for engulfment and with LST-4 and DYN-1 to promote phagosome maturation needed for corpse degradation. Given that the major factors for apoptotic cell engulfment are evolutionarily conserved and the interactions of clathrin and AP2 with CED-6 and/or CED-1 similarly exist in *C. elegans*, *Drosophila* and mammals, our discovery that clathrin and AP2 play an essential role in removal of apoptotic cells suggests that the non-classical function of clathrin and its adaptor proteins in phagocytosis is likely conserved across diverse species.

## Materials and Methods

### *C. elegans* strains and genetics

The Bristol strain N2 was used as wild type. *lst-4(tm2423)* deletion mutants were provided by Dr. Shohei Mitani (Tokyo Women's Medical University, Tokyo, Japan). *lst-4(qx159)* mutants were isolated in Dr. Xiaochen Wang's lab (National Institute of Biological Sciences, Beijing). The *lst-4(qx159)* mutation is a deletion of 4573 bp including 1962 bp of the *lst-4* gene (from exon 4 to the stop codon) and 2611 bp downstream of the *lst-4* open reading frame (ORF), which also affects the gene Y37A1B.4. The flanking sequences of the deletion region are 5′-TGCCCAGAAATTTTATTTTT-3′ and 5′-ATGTTCTTGTTGACCTTATT-3′. Other mutant alleles used in this study are listed by linkage groups: LG I: *ced-1(e1735)*, *ced-12(n3261)*. LG III: *ced-6(n1813)*, *ced-6(n2095)*, *chc-1(b1025ts)*. LG IV: *ced-2(n1994)*, *ced-5(n1812)*. LG V: *unc-76(e911)*. LG X: *dyn-1(ky51)*. The integrated arrays *qxIs405*(P*_ced-1_gfp::act-1*), *qxIs105* (P*_rab-14_ mcherry::rab-14*), *qxIs139* (derived from *qxEx957*) (P*_ced-1_dyn-1::gfp), qxIs408* (P*_ced-1_gfp::rab-5*), *qxIs66* (P*_ced-1_gfp::rab-7*), *qxIs257* (P*_ced-1_ nuc-1::mcherry*) were provided by Dr. Xiaochen Wang. The integrated arrays *smIs34* (P*_ced-1_ ced-1::gfp*) and *smIs110* (P*_ced-1_ ced-1*Δ*C::gfp*) were provided by Dr. Ding Xue (University of Colorado, Boulder). The integrated array *opIs334* (P*_ced-1_yfp::2×fyve*) was provided by Dr. K. S. Ravichandran (University of Virginia, Charlottesville, VA) and Dr. M. O. Hengartner (University of Zurich, Zurich, Switzerland). The integrated array *yqIs120* (P*_dpy-23_dpy-23::gfp*) was generated by integrating an extrachromosomal transgene harboring the pMG4 (P*_dpy-23_dpy-23::gfp*) plasmid kindly provided by Dr. Erik M. Jorgensen (University of Utah, Salt Lake City, UT) and the *unc-76* rescuing plasmid in an *unc-76(e911)* background. Other strains used in this study carrying integrated or extrachromosomal arrays are as follows: *yqIs98* (P*_ced-1_mCherry::chc-1*), *yqIs99* (P*_ced-1_apa-2::gfp*), *yqIs100* (P*_ced-1_mCherry::act-1*), *yqIs101* (P*_ced-1_gfp::ced-6*), *yqIs112* (P*_ced-1_gfp::chc-1*), *yqIs114* (P*_lst-4_lst-4*(cDNA)::*gfp*), *yqIs119* (P*_lst-4_lst-4*(cDNA)::*mCherry*), *yqIs121*(P*_ced-1_gfp::Moesin*), *yqEx368* (P*_lst-4_*::*lst-4*(cDNA)::*gfp*), *yqEx376* (P*_lst-4_*::*lst-4*(gDNA)::*gfp*), *yqEx480* (*P_chc-1_chc-1::gfp*), *yqEx481* (*P_apa-2_apa-2::gfp*). Animals carrying the stably integrated array were outcrossed with the N2 strain 4 times. *C. elegans* cultures and genetic crosses were performed essentially according to standard procedures [56]. Deletion strains were outcrossed with the N2 strain at least 4 times. *C. elegans* transformation was carried out essentially as described before [57].

### Plasmid construction

The P*_chc-1_chc-1::gfp* construct was generated by cloning a genomic DNA fragment containing a promoter region of 3 kb and the open reading frame (ORF) of the *chc-1* gene in frame with GFP into the pPD95.77 vector. The P*_apa-2_apa-2::gfp* construct was similarly generated by cloning a genomic fragment containing a promoter region of 2 kb and the ORF of *apa-2*. Genomic DNA containing the ORF of *chc-1* was amplified and inserted into P*_ced-1_mCherry*1 or P*_ced-1_gfp*1 via the KpnI site to generate the P*_ced-1_mCherry::chc-1* and P*_ced-1_gfp::chc-1* constructs. To generate P*_ced-1_apa-2::gfp*, a genomic fragment containing the *apa-2* ORF was amplified and inserted into P*_ced-1_gfp*3 via the KpnI site. To construct P*_ced-1_gfp::Moesin*, the C-terminal of Moesin was amplified from the plasmid pJWZ6 [58] (provided by Dr. David R. Sherwood, Duke University) and inserted into P*_ced-1_gfp*1 via the KpnI site. To generate P*_lst-4_lst-4*(cDNA)::*gfp*, a DNA fragment containing a 2 kb promoter region and the first intron followed by the remaining cDNA sequence of the *lst-4* isoform c was inserted between the HindIII and KpnI sites of the vector pPD95.77. P*_lst-4_lst-4*(cDNA)::*mCherry* was derived from P*_lst-4_lst-4*(cDNA)::*gfp* by replacing *gfp* with *mCherry*. To generate P*_lst-4_lst-4*(gDNA)::*gfp*, a genomic fragment containing a 2 kb promoter region and the *lst-4* genomic ORF were amplified and inserted between the XbaI and XmaI sites of the vector pPD95.77.

### RNAi experiments

RNAi experiments were performed by using bacterial feeding assays as described previously [59]. In most cases, L4-stage animals were transferred to plates seeded with bacteria expressing either control double-stranded RNA (dsRNA) (L4440 empty vector) (Control RNAi) or dsRNA corresponding to the open reading frames of genes of interest. RNAi of *apa-2* with its 3′ UTR was performed by feeding animals with bacteria expressing dsRNA corresponding to the 3′UTR of 516 bp. Germ cell corpses and other phenotypes were observed in adults of the progeny. For RNAi of *chc-1*, *apb-1* and *dyn-1*, which may cause embryonic lethality in the progeny, L3- to L4-stage animals were transferred to plates seeded with bacteria expressing dsRNA of individual genes and phenotypes were observed in adults of the same generation.

### Quantification of cell corpses

Cell corpses in synchronized animals were scored under Nomarski optics. To quantify germ cell corpses, cell corpses in the germline meiotic region of one gonad arm in each of at least 15 animals were scored at various adult ages (12, 24, 36, 48 and 60 h after the L4 stage). The average numbers of germ cell corpses from one gonad arm were calculated for each adult age. Data derived from different genetic backgrounds were compared using unpaired *t*-tests. For cell corpse analysis of *chc-1(b1025ts)* mutants, animals were grown to L4 at 20°C and then shifted to 25°C. Germ cell corpses were scored at 12, 24, 36 and 48 h after the shift.

### Immunofluorescence microscopy

To quantify the percentage of germ cell corpses labeled by various phagosomal markers, adult animals at 36 h after the L4 molt were mounted on agar pads in M9 buffer (1 litre contains: 3 g KH_2_PO_4_, 6 g Na_2_HPO_4_, 5 g NaCl, 1 mM MgSO_4_) with 2 mM levamisole and then examined by fluorescence microscopy. To analyze the labeling of germ cell corpse by phagosomal markers in *dyn-1(ky51ts)* mutants, animals were grown to L4 stage at 20°C and then shifted to 25°C; cell corpses were analyzed 24 h after the shift. To view germ cell corpses in *dyn-1* RNAi-treated animals, L4 larvae were cultured on RNAi plates and germ cell corpses were analyzed 24 h after the L4 molt.

### LysoSensor Green DND-189 staining

Adult animals (36 h after the L4 molt) were dissected in gonad dissection buffer (60 mM NaCl, 32 mM KCl, 3 mM Na_2_HPO_4_, 2 mM MgCl_2_, 20 mM Hepes, 50 µg/ml penicillin, 50 µg/ml streptomycin, 100 µg/ml neomycin, 10 mM glucose, 33% FCS, and 2 mM CaCl_2_) containing 1 µM LysoSensor Green DND-189 (Invitrogen) and examined by fluorescence microscopy.

### Time-lapse analysis of cell corpses

To measure the duration of germ cell corpses, animals were mounted in M9 buffer containing 2 mM levamisole, sealed with beeswax and Vaseline (1∶1), and recorded under Nomarski optics at 20°C. The gonadal region was recorded every 1 min at 1 µm/section for 20 *Z*-sections. Images were captured using a Zeiss Axioimager M1 coupled with an AxioCam monochrome digital camera and Axiovision rel. 4.7 software. Animals were constantly examined for viability during recording.

### Transmission electron microscopy (TEM) analysis

L3- or L4-stage animals were fed with bacteria expressing dsRNA of *chc-1* or *apb-1*. 30 h later, animals were collected for fixation, embedding and sectioning following a procedure essentially as described by Gumienny et al. [60]. Cell corpse photographs were taken with a JEM-1400 Transmission Electron Microscope. Germ cell corpses and the neighboring gonadal sheath cells were analyzed to determine whether individual cell corpses were engulfed.

### Recombinant proteins and GST pull down

Recombinant GST-CED-1C, GST-CED-6, GST-DYN-1, GST-LST-4, GST-CED-9 proteins were expressed in bacterial BL21(DE3) cells and purified with glutathione-Sepharose beads (Amersham) according to the instructions provided by the supplier. His6-tagged CHC-1C (amino acids 825–1682), LST-4, DYN-1 and mCherry were purified with Ni-NTA beads. ^35^S-labeled APA-2, APB-1 and DPY-23 were prepared by in vitro translation. Purified GST, GST-CED-9, GST-CED-1C or GST-CED-6 proteins (3 µg of each) immobilized on glutathione-Sepharose beads was incubated with ^35^S-labeled APA-2, APB-1, DPY-23 or CHC-1CHis6, LST-4His6 and DYN-1His6 at 4°C for ≥4 h and washed extensively. Bound proteins were resolved on sodium dodecyl sulfate (SDS) polyacrylamide gels (SDS-PAGE) and visualized by autoradiography or immunoblotting with anti-His6 antibody.

### Antibodies, immunoblotting, and immunoprecipitation

CED-1C antibody was generated previously [22]. CED-6 and mCherry antibodies were generated in guinea pigs or rabbits by injecting recombinant proteins. GFP polyclonal antibody (Catalog # E022200-02, rabbit) was purchased from EarthOx, LLC. (San Francisco, CA, USA). GFP monoclonal antibody (GFP(B-2):sc-9996, mouse) was purchased from Santa Cruz Biotechnology, Inc. Whole cell lysates were prepared from indicated strains and immunoprecipitations were performed essentially as described before [22] using individual antibodies. Precipitated proteins were resolved by SDS-PAGE and subjected to LC-MS analysis or detected with antibodies.

### Time-lapse imaging

Adult animals (24 h post L4 molt) were anesthetized with 0.1 mM levamisole in M9 buffer, mounted on 2% agar pads, and maintained at 22°C. Time-lapse images of DYN-1::GFP, LST-4::mCherry and LST-4::GFP were captured every 2 min by using an imaging system consisting of an Axiovert 200M microscope (Carl Zeiss MicroImaging, Inc.) equipped with a 100×, 1.45 N.A. objective, an EM CCD camera (Hamamatsu model, C9100-13), and the 488 nm and 561 nm lines of an Argon and Krypton laser attached to a spinning disk confocal scan head (Yokogawa CSU10 obtained from Solamere Inc.).

## Supporting Information

Figure S1 Detection of CHC-1 in proteins co-immunoprecipitated with CED-1 (A) and CED-6 (B). Immunopreciptations were performed with CED-1C (A) and CED-6 (B) antibodies on lysates of N2, *ced-1(e1735)*, and *ced-6(n1813)* animals. The precipitated proteins were resolved on SDS-PAGEs and visualized with silver staining. Gel regions indicated by the brackets were cut off and subjected to mass spectrometry analysis by using a TripleTOF5600 mass spectrometer (AB Sciex, Canada). Protein identification was performed by searching the *C. elegans* proteome sequence database (SwissProt) using ProteinPilot software 4.2, with a mass tolerance of 0.05 Da and a false discovery rate of 1%. The unique peptides identified for CHC-1, CED-1, or CED-6 are shown on the right. Asterisks indicate the precipitated CED-1 (A) or CED-6 (B). Arrowheads indicate the IgG heavy chain.
(JPG)

Figure S2 Clathrin and AP2 associate with phagosomes containing cell corpses. (A) Representative DIC and fluorescence images of germ cell corpses surrounded by CHC-1::GFP, APA-2::GFP, and DPY-23::GFP in animals expressing P*_chc-1_chc-1::gfp*, P*_apa-2_apa-2::gfp*, and P*_dpy-23_dpy-23::gfp*, respectively. Arrows indicate cell corpses. Bars, 10 µm. (B) Rescue of the cell corpse phenotype in *chc-1(b1025ts)* mutants by P*_chc-1_chc-1*::*gfp* (*yqEx480*) and P*_ced-1_mCherry::chc-1* (*yqIs98*). (C) Rescue of the cell corpse phenotype in animals with RNAi of the 3′UTR of *apa-2* by P*_apa-2_apa-2::gfp* (*yqEx481*) and P*_ced-1_apa-2::gfp* (*yqIs99*). Germ cell corpses in one gonad arm were scored in each animal of the indicated strains at 36 h and 48 h post L4 stage. Error bars represent SEM. Comparisons were performed using unpaired *t*-tests. ** *p*<0.001. (D) Rescue of the cell corpse phenotype in *ced-1(e1735)* mutants by P*_ced-1_ced-1::gfp* (*smIs34*) and P*_ced-1_ced-1ΔC::gfp* (*smIs110*). (E) Rescue of the cell corpse phenotype in *ced-6(n2095)* mutants by P*_ced-1_gfp::ced-6* (*yqIs101*). In (D) and (E), the average number of cell corpses per embryo in 4-fold embryos (>15 embryos in total) and the average number of germ cell corpses per gonad arm (>20 germline arms in total) are shown for each genotype. Comparisons were performed with unpaired *t*-tests. ** *p*<0.001.
(JPG)

Figure S3 Loss of clathrin, AP2 and *lst-4* does not affect the encircling of germ cell corpses by CED-1::GFP and GFP::CED-6. (A and B) Representative images of germ cell corpses labeled by CED-1::GFP (A) or GFP::CED-6 (B) in N2, *apb-1(RNAi)*, *chc-1(RNAi)* and *lst-4(tm2423)* animals. Arrows point to cell corpses labeled by CED-1::GFP or GFP::CED-6; arrowheads indicate unlabeled corpses. Bars, 10 µm. (C) Quantification of cell corpse labeling by CED-1::GFP and GFP::CED-6 in the animals indicated. ≥100 corpses were analyzed for each genotype.
(JPG)

Figure S4 CHC-1 and AP2 are required for the rearrangement of the actin cytoskeleton. (A) Representative images of cell corpse labeling by GFP::Moesin in *Ctrl(RNAi)*, *ced-1(RNAi)*, *ced-6(RNAi)*, *chc-1(RNAi)* and *apb-1(RNAi)* germ lines. Bars, 10 µm. (B) Quantification of the labeling of germ cell corpses by GFP::Moesin as shown in (A). ≥100 corpses were scored for each genotype.
(JPG)

Figure S5 LST-4 affects phagosomal recruitment of factors required for phagosome maturation. (A) Schematic representation of the *lst-4(tm2423)* and *lst-4(qx159)* deletion mutation. Solid boxes indicate exons and thin lines indicate introns. Deleted regions are indicated by the bars above and below the gene. (B) Quantification of germ cell corpses in N2, *lst-4(tm2423)* and *lst-4(qx159)*. Error bars represent SEM. N2 and *lst-4* mutants were compared using unpaired *t*-tests. ** *p*<0.001. (C–G) Representative DIC and fluorescence images of germ cell corpse labeling by GFP:Moesin (C), YFP::2XFYVE (D), GFP::RAB-5 (E), mCherry::RAB-14 (F) and GFP::RAB-7 (G) in N2 and *lst-4(tm2423)* mutants. Arrows indicate cell corpses labeled by phagosomal markers and arrowheads indicate unlabeled corpses. Bars, 10 µm. (H) Quantification of germ cell corpse labeling as shown in (C–G). The data represent average numbers of 3 independent experiments. ≥100 corpses were scored for each phagosomal marker at each time. Error bars represent SEM.
(JPG)

Figure S6 Characterization of LST-4-mediated phagosome acidification. (A) Representative DIC and fluorescence images of cell corpse staining by LysoSensor Green DND-189 in *gla-3(RNAi)*, *lst-4(tm2423)*, *vps-18(tm1125)* and *vps-18(tm1125);lst-4(tm2423)* germ lines. Arrows point to germ cell corpses positive for LysoSensor Green DND-189; arrowheads indicate unstained corpses. Bars, 10 µm. (B) Quantification of cell corpse staining as shown in (A). ≥100 corpses were scored for each genotype. (C) Expression and localization of LST-4::GFP driven by the *lst-4* promoter. The transgenic array used is *yqEx376* (P*_lst-4_*::*lst-4*(gDNA)::*gfp*). LST-4::GFP is observed in ∼100-cell stage embryos and in adult gonadal sheath cells. Bars, 10 µm. (D) Rescue of the cell corpse phenotype in *lst-4(tm2423)* mutants by P*_lst-4_*::*lst-4*(gDNA)::*gfp* (*yqEx376*), P*_lst-4_*::*lst-4*(cDNA)::*gfp* (*yqEx368*), and P*_lst-4_*::*lst-4*(cDNA)::*mCherry* (*yqIs119*). Germ cell corpses in one gonad arm were scored in each animal of the indicated strains at 36 h post L4 stage. For transgenes, the number of cell corpses in one transgenic line from a total of three lines exhibiting similar rescuing activity is shown. Error bars represent SEM. Comparisons were performed between *lst-4(tm2423)* and transgenic animals by unpaired *t*-tests. ** *p*<0.001. (E and F) Representative DIC and fluorescence images of germ cell corpse labeling by LST-4::GFP (*yqIs114*) in mutants affecting engulfment (E) and animals with RNAi of genes required for phagosome maturation (F). Arrows indicate cell corpses labeled by LST-4::GFP and arrowheads indicate unlabeled corpses. Bars, 10 µm.
(JPG)

Figure S7 LST-4 interacts with DYN-1 and acts downstream of clathrin and AP2. (A) DYN-1::GFP and LST-4::mCherry colocalize on the surface of germ cell corpses. DIC, GFP, mCherry and merged GFP and mCherry images are shown. Cell corpses are indicated by arrows. Bar, 10 µm. (B) Rescue of the cell corpse phenotype in *dyn-1(ky51)* mutants by P*_ced-1_dyn-1::gfp* (*qxEx957*). Germ cell corpse were scored at 24 and 36 h post L4 stage and analyzed as in Figure S6D. (C) LST-4 and DYN-1 interact with one other. In the top panel, IPs were performed using mCherry antibody on lysates of animals expressing both LST-4::mCherry and DYN-1::GFP and animals expressing DYN-1::GFP alone. Precipitated proteins were detected with GFP and mCherry antibodies. In the upper part of the bottom panel, purified GST, GST-CED-1C, GST-CED-6 and GST-DYN-1 proteins (3 µg of each) immobilized on glutathione-Sepharose beads were incubated with LST-4His6 at 4°C for ≥4 h and washed extensively. Bound proteins were resolved on an SDS-PAGE and detected by anti-His6 antibody. In the lower part of the bottom panel, the interactions of DYN-1His6 protein with purified GST, GST-CED-1C and GST-CED-6 were examined as above. (D) Quantification of phagosomal association of LST-4::GFP and DYN-1::GFP in *Ctrl(RNAi)*, *apb-1(RNAi)* and *chc-1(RNAi)* animals as shown in Figure 7B and 7C. ≥100 corpses were analyzed for each genotype. (E-F) Representative images of phagosomal association of APA-2::GFP in N2, *lst-4(tm2423)* and *dyn-1(ky51)*(25°C) germ lines (E) and phagosomal association of mCherry::CHC-1 in *gla-3(RNAi)*, *lst-4(tm2423)*, and *dyn-1(RNAi)* germ lines (F). Adult animals (24 h after the L4 molt) were analyzed. Arrows indicate cell corpses labeled by APA-2::GFP or mCherry::CHC-1. Bars, 10 µm. (G) Quantification of phagosomal association of APA-2::GFP as shown in (E) and *chc-1(RNAi)* germ lines (left) and phagosomal association of mCherry::CHC-1 as shown in (F) and *apb-1(RNAi)* germ lines (right). ≥100 corpses were analyzed for each genotype.
(JPG)

Table S1 Cell corpse phenotype caused by RNAi of *C. elegans* genes involved in clathrin-mediated endocytosis. *C. elegans* genes involved in clathrin-mediated endocytosis were identified by using sequences of individual human proteins to search for homologs in the *C. elegans* genome database. RNAi was performed as described in Methods. Germ cell corpses in one gonad arm of each animal were scored for at least 15 animals 60 h after the L4 stage. N/A indicates that *hsp-1* RNAi caused defects in germline proliferation and no cell corpses could be scored.
(DOC)
