# Supplementary figures and images for: Clathrin and AP2 Are Required for Phagocytic Receptor-Mediated Apoptotic Cell Clearance in Caenorhabditis elegans
Source: PLoS Genet. 2013 May 16;9(5):e1003517. doi: 10.1371/journal.pgen.1003517 (PMC3656144; doi:10.1371/journal.pgen.1003517)

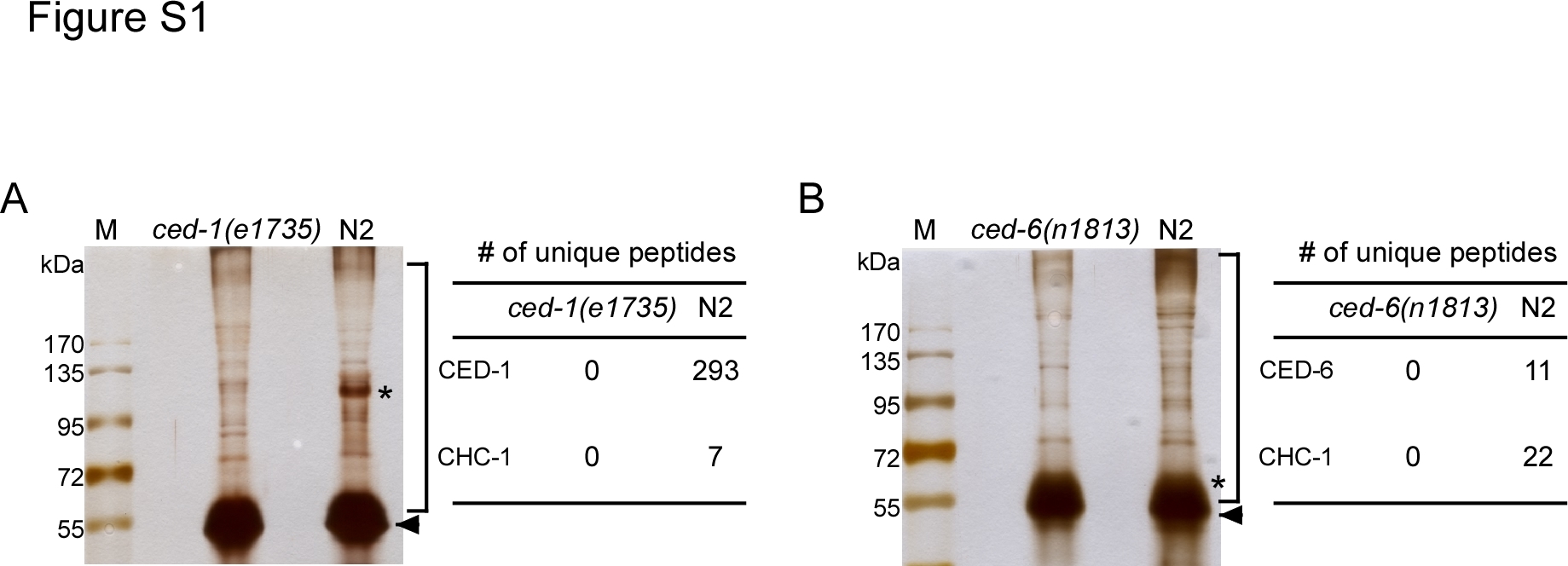

Supplement: Figure S1 — Detection of CHC-1 in proteins co-immunoprecipitated with CED-1 (A) and CED-6 (B). Immunopreciptations were performed with CED-1C (A) and CED-6 (B) antibodies on lysates of N2, ced-1(e1735), and ced-6(n1813) animals. The precipitated proteins were resolved on SDS-PAGEs and visualized with silver staining. Gel regions indicated by the brackets were cut off and subjected to mass spectrometry analysis by using a TripleTOF5600 mass spectrometer (AB Sciex, Canada). Protein identification was performed by searching the C. elegans proteome sequence database (SwissProt) using ProteinPilot software 4.2, with a mass tolerance of 0.05 Da and a false discovery rate of 1%. The unique peptides identified for CHC-1, CED-1, or CED-6 are shown on the right. Asterisks indicate the precipitated CED-1 (A) or CED-6 (B). Arrowheads indicate the IgG heavy chain. (JPG) [file pgen.1003517.s001.jpg]

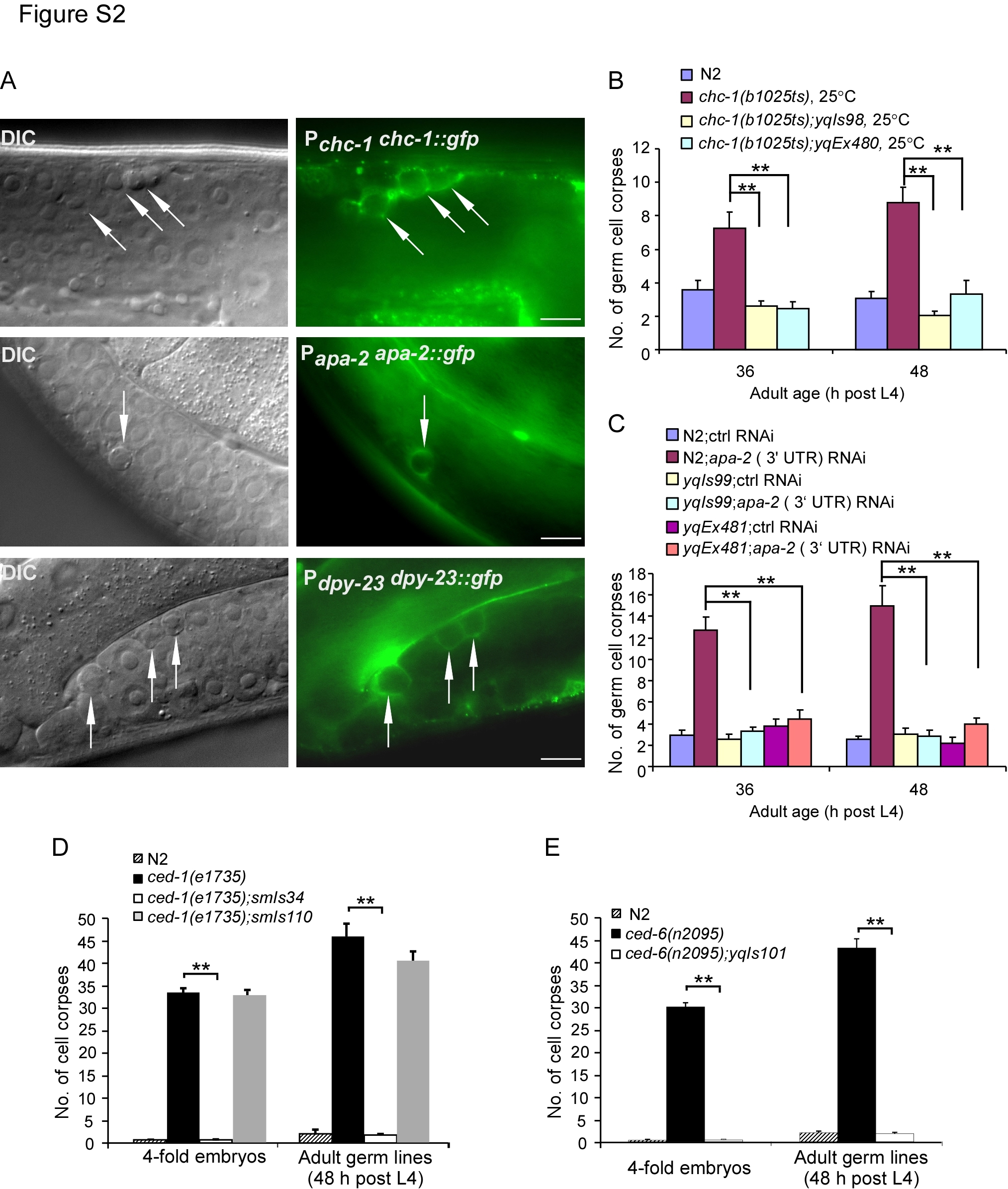

Supplement: Figure S2 — Clathrin and AP2 associate with phagosomes containing cell corpses. (A) Representative DIC and fluorescence images of germ cell corpses surrounded by CHC-1::GFP, APA-2::GFP, and DPY-23::GFP in animals expressing Pchc-1chc-1::gfp, Papa-2apa-2::gfp, and Pdpy-23dpy-23::gfp, respectively. Arrows indicate cell corpses. Bars, 10 µm. (B) Rescue of the cell corpse phenotype in chc-1(b1025ts) mutants by Pchc-1chc-1::gfp (yqEx480) and Pced-1mCherry::chc-1 (yqIs98). (C) Rescue of the cell corpse phenotype in animals with RNAi of the 3′UTR of apa-2 by Papa-2apa-2::gfp (yqEx481) and Pced-1apa-2::gfp (yqIs99). Germ cell corpses in one gonad arm were scored in each animal of the indicated strains at 36 h and 48 h post L4 stage. Error bars represent SEM. Comparisons were performed using unpaired t-tests. ** p<0.001. (D) Rescue of the cell corpse phenotype in ced-1(e1735) mutants by Pced-1ced-1::gfp (smIs34) and Pced-1ced-1ΔC::gfp (smIs110). (E) Rescue of the cell corpse phenotype in ced-6(n2095) mutants by Pced-1gfp::ced-6 (yqIs101). In (D) and (E), the average number of cell corpses per embryo in 4-fold embryos (>15 embryos in total) and the average number of germ cell corpses per gonad arm (>20 germline arms in total) are shown for each genotype. Comparisons were performed with unpaired t-tests. ** p<0.001. (JPG) [file pgen.1003517.s002.jpg]

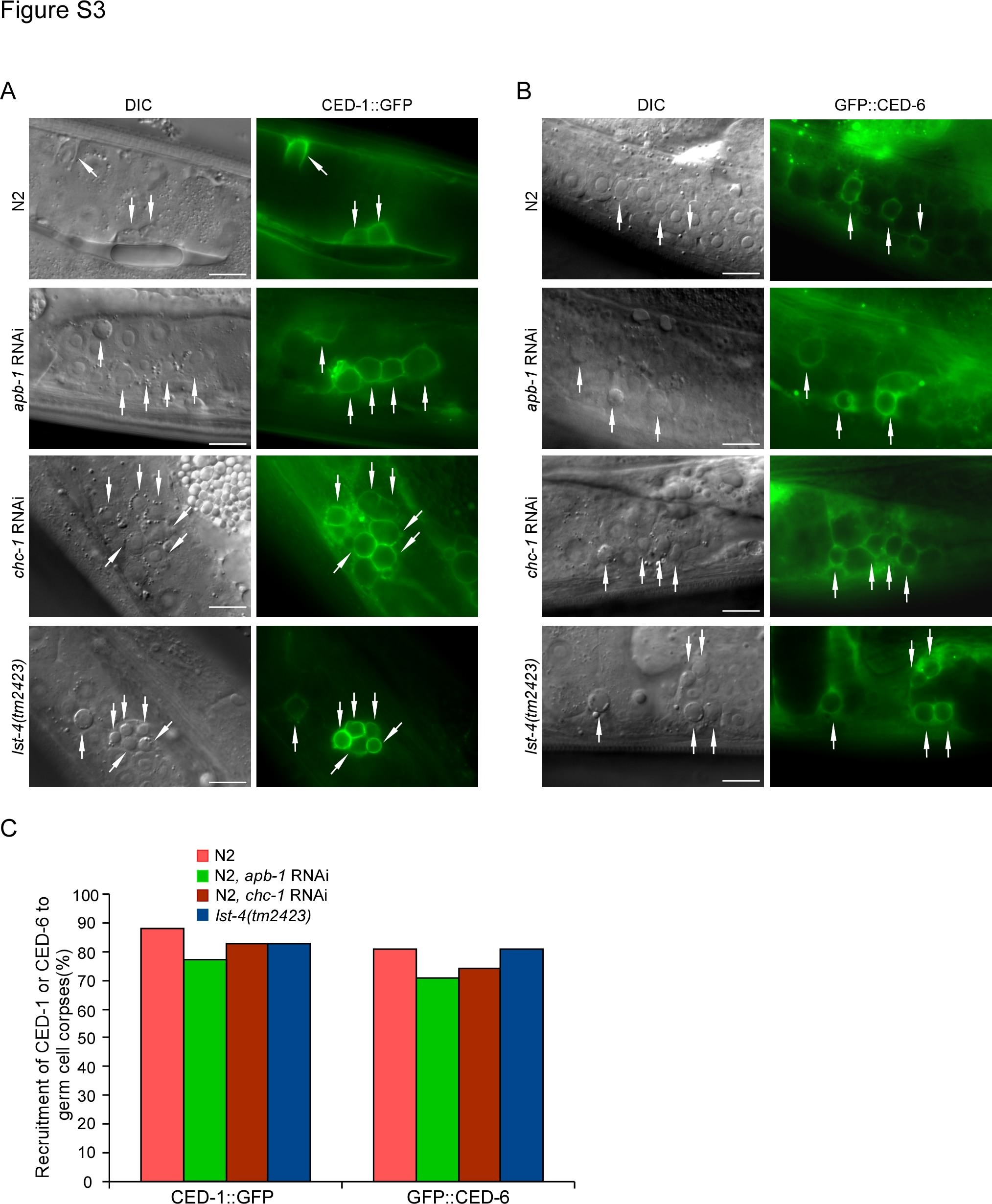

Supplement: Figure S3 — Loss of clathrin, AP2 and lst-4 does not affect the encircling of germ cell corpses by CED-1::GFP and GFP::CED-6. (A and B) Representative images of germ cell corpses labeled by CED-1::GFP (A) or GFP::CED-6 (B) in N2, apb-1(RNAi), chc-1(RNAi) and lst-4(tm2423) animals. Arrows point to cell corpses labeled by CED-1::GFP or GFP::CED-6; arrowheads indicate unlabeled corpses. Bars, 10 µm. (C) Quantification of cell corpse labeling by CED-1::GFP and GFP::CED-6 in the animals indicated. ≥100 corpses were analyzed for each genotype. (JPG) [file pgen.1003517.s003.jpg]

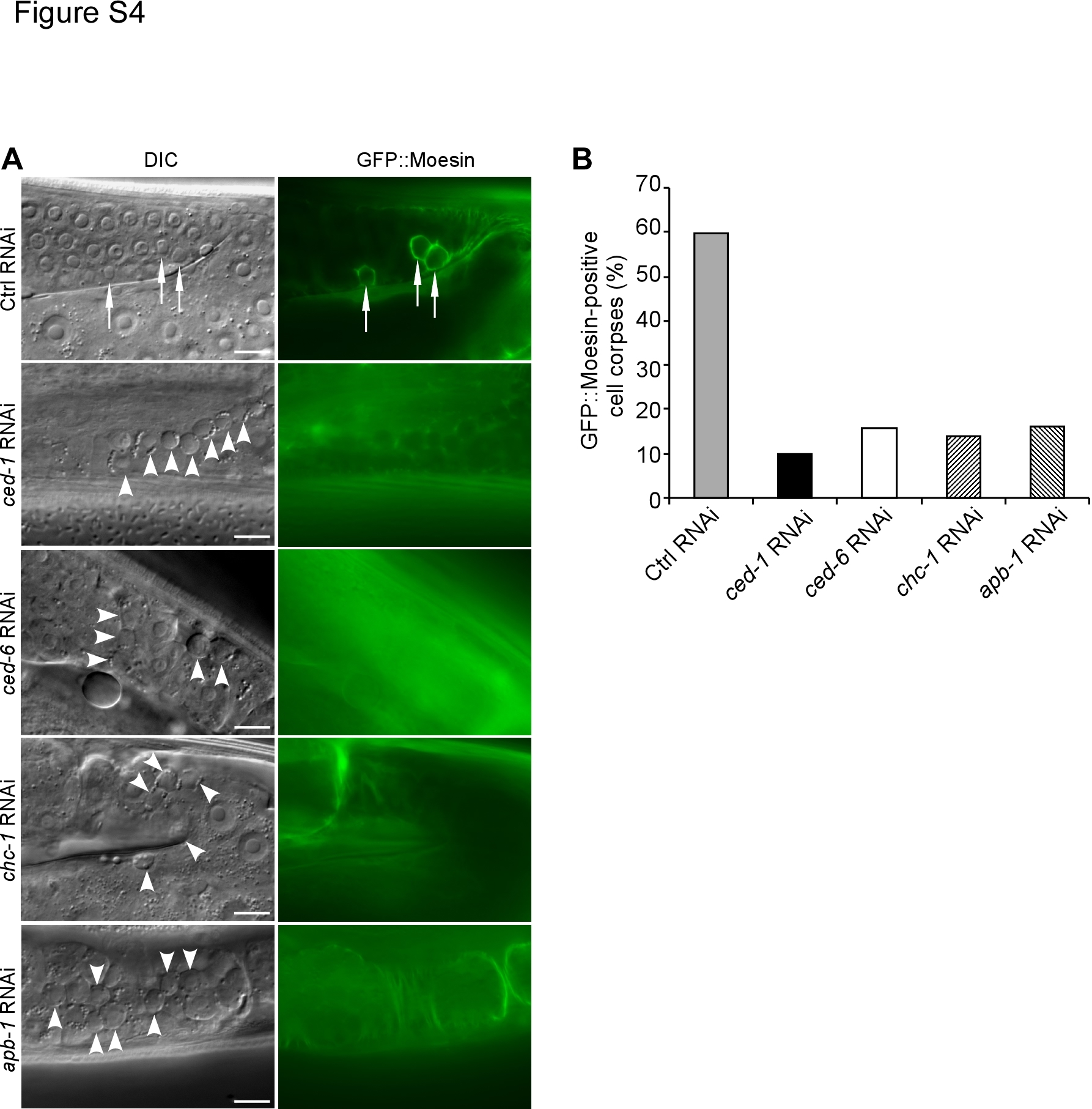

Supplement: Figure S4 — CHC-1 and AP2 are required for the rearrangement of the actin cytoskeleton. (A) Representative images of cell corpse labeling by GFP::Moesin in Ctrl(RNAi), ced-1(RNAi), ced-6(RNAi), chc-1(RNAi) and apb-1(RNAi) germ lines. Bars, 10 µm. (B) Quantification of the labeling of germ cell corpses by GFP::Moesin as shown in (A). ≥100 corpses were scored for each genotype. (JPG) [file pgen.1003517.s004.jpg]

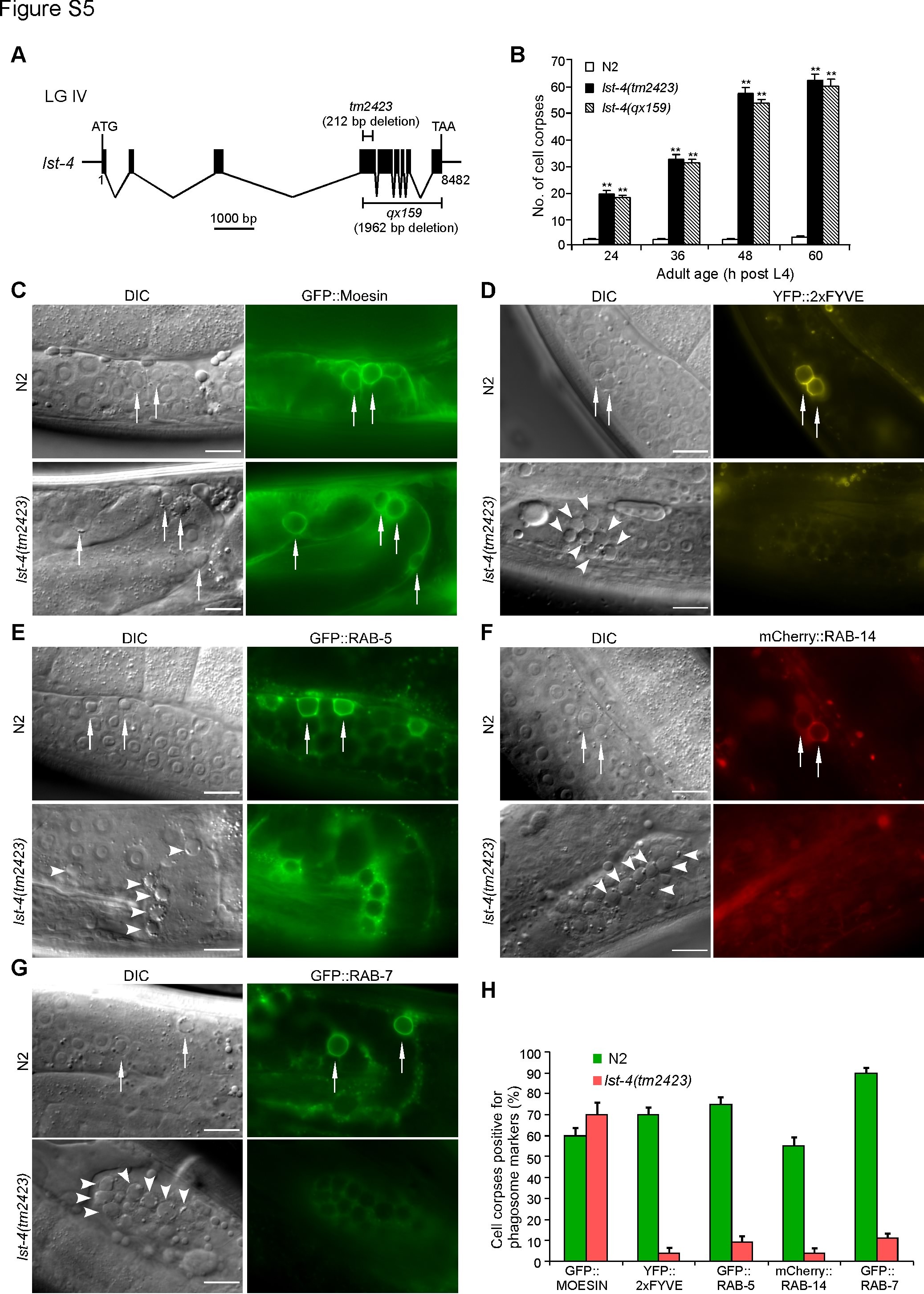

Supplement: Figure S5 — LST-4 affects phagosomal recruitment of factors required for phagosome maturation. (A) Schematic representation of the lst-4(tm2423) and lst-4(qx159) deletion mutation. Solid boxes indicate exons and thin lines indicate introns. Deleted regions are indicated by the bars above and below the gene. (B) Quantification of germ cell corpses in N2, lst-4(tm2423) and lst-4(qx159). Error bars represent SEM. N2 and lst-4 mutants were compared using unpaired t-tests. ** p<0.001. (C–G) Representative DIC and fluorescence images of germ cell corpse labeling by GFP:Moesin (C), YFP::2XFYVE (D), GFP::RAB-5 (E), mCherry::RAB-14 (F) and GFP::RAB-7 (G) in N2 and lst-4(tm2423) mutants. Arrows indicate cell corpses labeled by phagosomal markers and arrowheads indicate unlabeled corpses. Bars, 10 µm. (H) Quantification of germ cell corpse labeling as shown in (C–G). The data represent average numbers of 3 independent experiments. ≥100 corpses were scored for each phagosomal marker at each time. Error bars represent SEM. (JPG) [file pgen.1003517.s005.jpg]

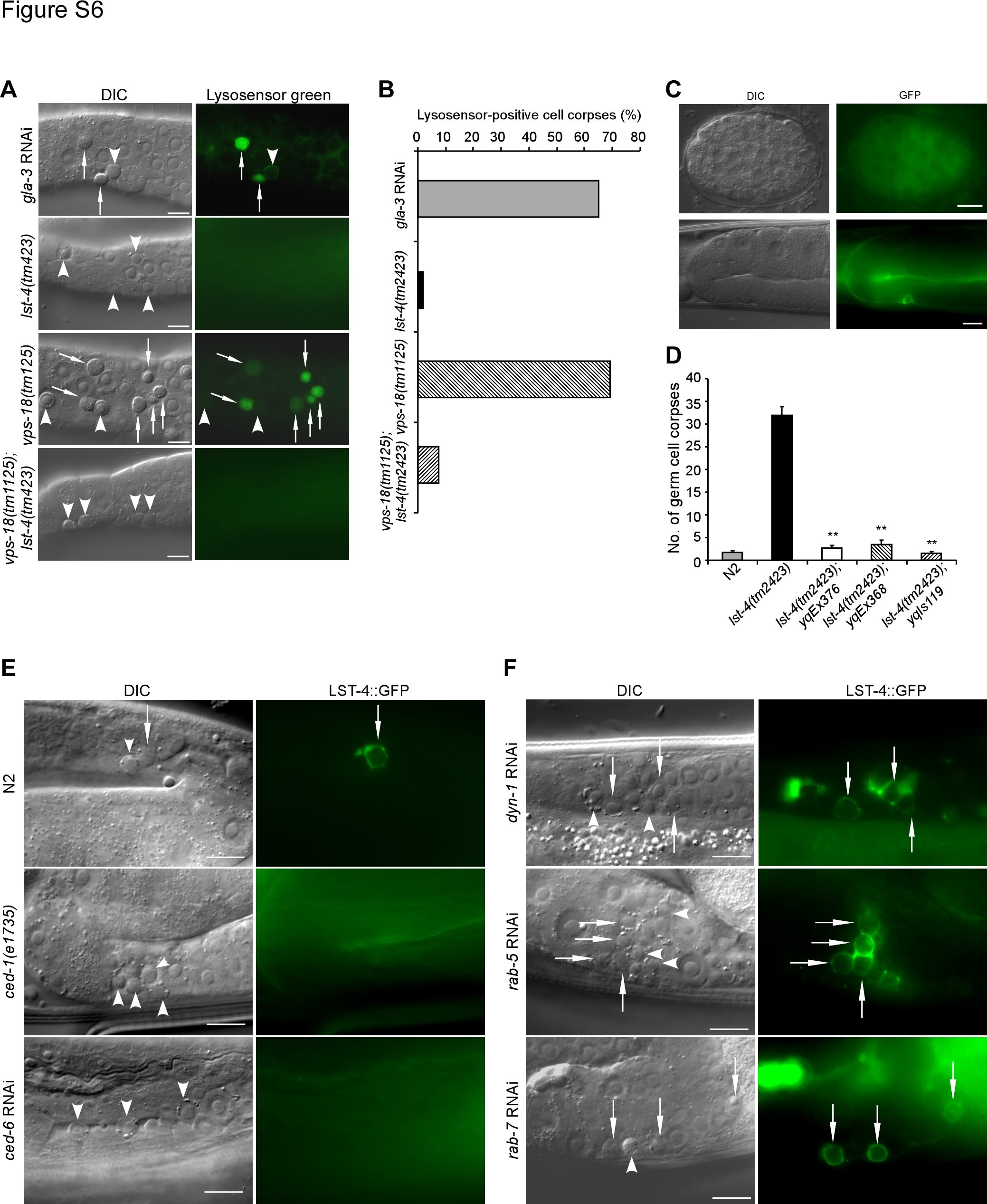

Supplement: Figure S6 — Characterization of LST-4-mediated phagosome acidification. (A) Representative DIC and fluorescence images of cell corpse staining by LysoSensor Green DND-189 in gla-3(RNAi), lst-4(tm2423), vps-18(tm1125) and vps-18(tm1125);lst-4(tm2423) germ lines. Arrows point to germ cell corpses positive for LysoSensor Green DND-189; arrowheads indicate unstained corpses. Bars, 10 µm. (B) Quantification of cell corpse staining as shown in (A). ≥100 corpses were scored for each genotype. (C) Expression and localization of LST-4::GFP driven by the lst-4 promoter. The transgenic array used is yqEx376 (Plst-4::lst-4(gDNA)::gfp). LST-4::GFP is observed in ∼100-cell stage embryos and in adult gonadal sheath cells. Bars, 10 µm. (D) Rescue of the cell corpse phenotype in lst-4(tm2423) mutants by Plst-4::lst-4(gDNA)::gfp (yqEx376), Plst-4::lst-4(cDNA)::gfp (yqEx368), and Plst-4::lst-4(cDNA)::mCherry (yqIs119). Germ cell corpses in one gonad arm were scored in each animal of the indicated strains at 36 h post L4 stage. For transgenes, the number of cell corpses in one transgenic line from a total of three lines exhibiting similar rescuing activity is shown. Error bars represent SEM. Comparisons were performed between lst-4(tm2423) and transgenic animals by unpaired t-tests. ** p<0.001. (E and F) Representative DIC and fluorescence images of germ cell corpse labeling by LST-4::GFP (yqIs114) in mutants affecting engulfment (E) and animals with RNAi of genes required for phagosome maturation (F). Arrows indicate cell corpses labeled by LST-4::GFP and arrowheads indicate unlabeled corpses. Bars, 10 µm. (JPG) [file pgen.1003517.s006.jpg]

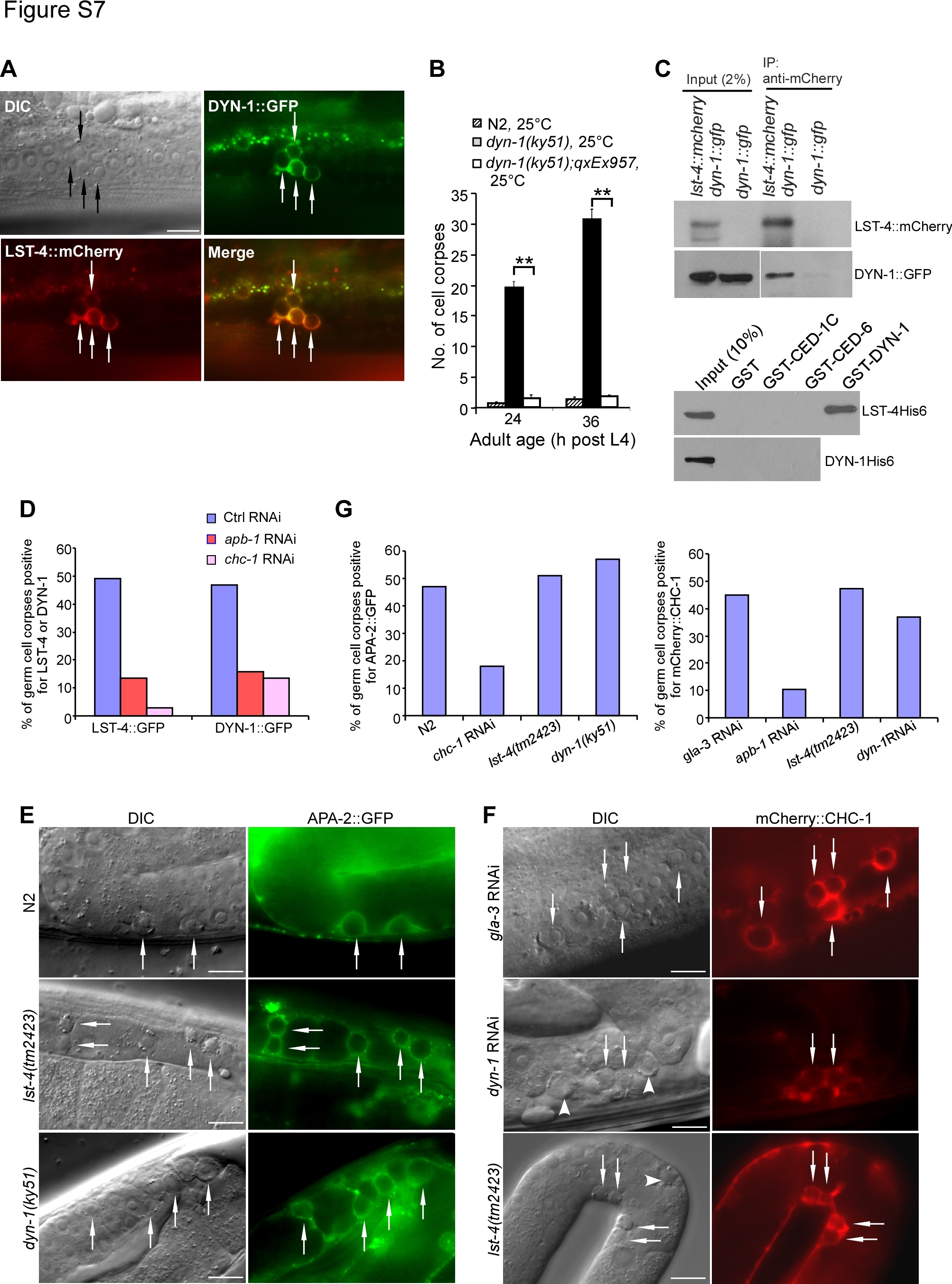

Supplement: Figure S7 — LST-4 interacts with DYN-1 and acts downstream of clathrin and AP2. (A) DYN-1::GFP and LST-4::mCherry colocalize on the surface of germ cell corpses. DIC, GFP, mCherry and merged GFP and mCherry images are shown. Cell corpses are indicated by arrows. Bar, 10 µm. (B) Rescue of the cell corpse phenotype in dyn-1(ky51) mutants by Pced-1dyn-1::gfp (qxEx957). Germ cell corpse were scored at 24 and 36 h post L4 stage and analyzed as in Figure S6D. (C) LST-4 and DYN-1 interact with one other. In the top panel, IPs were performed using mCherry antibody on lysates of animals expressing both LST-4::mCherry and DYN-1::GFP and animals expressing DYN-1::GFP alone. Precipitated proteins were detected with GFP and mCherry antibodies. In the upper part of the bottom panel, purified GST, GST-CED-1C, GST-CED-6 and GST-DYN-1 proteins (3 µg of each) immobilized on glutathione-Sepharose beads were incubated with LST-4His6 at 4°C for ≥4 h and washed extensively. Bound proteins were resolved on an SDS-PAGE and detected by anti-His6 antibody. In the lower part of the bottom panel, the interactions of DYN-1His6 protein with purified GST, GST-CED-1C and GST-CED-6 were examined as above. (D) Quantification of phagosomal association of LST-4::GFP and DYN-1::GFP in Ctrl(RNAi), apb-1(RNAi) and chc-1(RNAi) animals as shown in Figure 7B and 7C. ≥100 corpses were analyzed for each genotype. (E-F) Representative images of phagosomal association of APA-2::GFP in N2, lst-4(tm2423) and dyn-1(ky51)(25°C) germ lines (E) and phagosomal association of mCherry::CHC-1 in gla-3(RNAi), lst-4(tm2423), and dyn-1(RNAi) germ lines (F). Adult animals (24 h after the L4 molt) were analyzed. Arrows indicate cell corpses labeled by APA-2::GFP or mCherry::CHC-1. Bars, 10 µm. (G) Quantification of phagosomal association of APA-2::GFP as shown in (E) and chc-1(RNAi) germ lines (left) and phagosomal association of mCherry::CHC-1 as shown in (F) and apb-1(RNAi) germ lines (right). ≥100 corpses were analyzed for each genoty [file pgen.1003517.s007.jpg]
