# Supplementary material for: Clathrin and AP2 Are Required for Phagocytic Receptor-Mediated Apoptotic Cell Clearance in Caenorhabditis elegans
Source: PLoS Genet. 2013 May 16;9(5):e1003517. doi: 10.1371/journal.pgen.1003517 (PMC3656144; doi:10.1371/journal.pgen.1003517)
Supplement: Table S1 — Cell corpse phenotype caused by RNAi of C. elegans genes involved in clathrin-mediated endocytosis. C. elegans genes involved in clathrin-mediated endocytosis were identified by using sequences of individual human proteins to search for homologs in the C. elegans genome database. RNAi was performed as described in Methods. Germ cell corpses in one gonad arm of each animal were scored for at least 15 animals 60 h after the L4 stage. N/A indicates that hsp-1 RNAi caused defects in germline proliferation and no cell corpses could be scored. (DOC) [file pgen.1003517.s008.doc]

**Table S1**

Mammalian *C. elegans* genes  No. of germ cell corpses

homologs (RNAi) (Mean ± SEM)

Control RNAi 1.2±0.3

Clathrin heavy chain *chc-1* ([*T20G5.1*](http://www.wormbase.org/db/seq/sequence?name=T20G5.1;class=Gene_name)) 13.3±0.7

Clathrin light chain *clic-1* ([*T05B11.3*](http://www.wormbase.org/db/seq/sequence?name=T05B11.3;class=Gene_name)) 0.7± 0.5

AP2 α subunit *apa-2* ([*T20B5.1*](http://www.wormbase.org/db/seq/sequence?name=T20B5.1;class=Gene_name)) 7.9 ± 0.6

AP2 β2 subunit *apb-1* ([*Y71H2B.10*](http://www.wormbase.org/db/seq/sequence?name=Y71H2B.10;class=Gene_name)) 18.6±1.5

AP2 μ2 subunit *dpy-23* (*R160.1*) 7.1±1.2

AP2 σ subunit *aps-2* (*F02E8.3*) 3.1±0.3

FCHo1/2 *F56D12.6* 4.2±0.2

Epsin1 *epn-1* ([*T04C10.2*](http://www.wormbase.org/db/seq/sequence?name=T04C10.2;class=Gene_name)) 2.1±0.4

Epsin4 *rsd-3* ([*C34E11.1*](http://www.wormbase.org/db/seq/sequence?name=C34E11.1;class=Gene_name)) 1.1±0.2

β-arrestin-1, 2 *arr-1* ([*F53H8.2*](http://www.wormbase.org/db/seq/sequence?name=F53H8.2;class=Gene_name)) 1.1±0.3

Dab2 *dab-1* ([*M110.5*](http://www.wormbase.org/db/seq/sequence?name=M110.5;class=Gene_name)) 4.5±0.3

Numb *num-1* ([*T03D8.1*](http://www.wormbase.org/db/seq/sequence?name=T03D8.1;class=Gene_name)) 1.9±0.1

AP180 *unc-11* ([*C32E8.10*](http://www.wormbase.org/db/seq/sequence?name=C32E8.10;class=Gene_name)) 1.9±0.2

AAK1  *sel-5(F35G12.3)*  2.1±0.6

*tag-257(F46G11.3)*  1.8±0.5

Endophilin *erp-1(F35A5.8)* 1.3±0.3

*unc-57(T04D1.3)* 1.1±0.3

HIP1 *tag-138(F08A8.6)* 3.1±1.2

HIP1R *hipr-1(ZK370.3)* 1.9±0.3

DYRK1A *mbk-1(T04C10.1)*  2.5±0.6

*mbk-2(F49E11.1)* 3.7±0.8

CVAK104 *ZC581.9* 1.2±0.3

Dynamin *dyn-1* ([*C02C6.1*](http://www.wormbase.org/db/seq/sequence?name=C02C6.1;class=Gene_name)) 37.7±1.2

Amphiphysin *amph-1* ([*F58G6.1*](http://www.wormbase.org/db/seq/sequence?name=F58G6.1;class=Gene_name)) 1.9±0.1

Eps15 *ehs-1* ([*ZK1248.3*](http://www.wormbase.org/db/seq/sequence?name=ZK1248.3;class=Gene_name)) 3.4±0.2

NECAP1, 2 *Y110A2AR.3* 5.4±0.3

Intersectin1, 2 *itsn-1* ([*Y116A8C.36*](http://www.wormbase.org/db/seq/sequence?name=Y116A8C.36;class=Gene_name)) 3.6±0.2

Stonin1, 2 *unc-41* ([*C27H6.1*](http://www.wormbase.org/db/seq/sequence?name=C27H6.1;class=Gene_name)) 1.9±0.3

Synaptojanin 1 *unc-26* ([*JC8.10*](http://www.wormbase.org/db/seq/sequence?name=JC8.10;class=Gene_name)) 1.6±0.2

Auxilin *dnj-25* ([*W07A8.3*](http://www.wormbase.org/db/seq/sequence?name=W07A8.3;class=Gene_name)) 1.7±0.2

Hsc70 *hsp-1* ([*F26D10.3*](http://www.wormbase.org/db/seq/sequence?name=F26D10.3;class=Gene_name)) N/A
